# Mapping of QTLs for Yield Traits Using F_2:3:4_ Populations Derived From Two Alien Introgression Lines Reveals *qTGW8.1* as a Consistent QTL for Grain Weight From *Oryza nivara*

**DOI:** 10.3389/fpls.2022.790221

**Published:** 2022-03-09

**Authors:** Kavitha Beerelli, Divya Balakrishnan, Krishnam Raju Addanki, Malathi Surapaneni, Venkateswara Rao Yadavalli, Sarla Neelamraju

**Affiliations:** ^1^National Professor Project, ICAR-Indian Institute of Rice Research, Hyderabad, India; ^2^Department of Biotechnology, Acharya Nagarjuna University, Guntur, India

**Keywords:** *Oryza*, alien introgression lines, yield QTL, thousand grain weight, wild species

## Abstract

Wild introgressions play a crucial role in crop improvement by transferring important novel alleles and broadening allelic diversity of cultivated germplasm. In this study, two stable backcross alien introgression lines 166s and 14s derived from Swarn/*Oryza nivara* IRGC81848 were used as parents to generate populations to map quantitative trait loci (QTLs) for yield-related traits. Field evaluation of yield-related traits in F_2_, F_3_, and F_4_ population was carried out in normal irrigated conditions during the wet season of 2015 and dry seasons of 2016 and 2018, respectively. Plant height, tiller number, productive tiller number, total dry matter, and harvest index showed a highly significant association to single plant yield in F_2_, F_3_, and F_4_. In all, 21, 30, and 17 QTLs were identified in F_2_, F_2:3_, and F_2:4_, respectively, for yield-related traits. QTLs *qPH6.1* with 12.54% phenotypic variance (PV) in F_2_, *qPH1.1* with 13.01% PV, *qTN6.1* with 10.08% PV in F_2:3_, and *qTGW6.1* with 15.19% PV in F_2:4_ were identified as major effect QTLs. QTLs *qSPY4.1* and *qSPY6.1* were detected for grain yield in F_2_ and F_2:3_ with PV 8.5 and 6.7%, respectively. The trait enhancing alleles of QTLs *qSPY4.1*, *qSPY6.1, qPH1.1*, *qTGW6.1*, *qTGW8.1, qGN4.1*, and *qTDM5.1* were from *O. nivara*. QTLs of the yield contributing traits were found clustered in the same chromosomal region. *qTGW8.1* was identified in a 2.6 Mb region between RM3480 and RM3452 in all three generations with PV 6.1 to 9.8%. This stable and consistent *qTGW8.1* allele from *O. nivara* can be fine mapped for identification of causal genes. From this population, lines C_2_12, C_2_124, C_2_128, and C_2_143 were identified with significantly higher SPY and C_2_103, C_2_116, and C_2_117 had consistently higher thousand-grain weight values than both the parents and Swarna across the generations and are useful in gene discovery for target traits and further crop improvement.

## Introduction

Rice is one of three major food crops across the world especially in the most populated regions, and it provides up to 23% of calories for human consumption (Fisher et al., 2000). A total of 503.5 million tonnes (milled basis) of rice was consumed around the globe during 2017–18 (FAO et al., 2017). Rice breeders have major challenges in increasing the yield potential of the cultivars as there is stagnation due to narrow genetic diversity available in cultivated germplasm. Wild genetic material is a source of important alleles or genes for agronomic traits including yield. Most of the earlier studies using conventional plant breeding methods helped increase of the yield levels of rice by improving the related traits (Brondani et al., 2002). Wild rice species are more diverse in physiological, morphological, and agronomical characteristics than the existing cultivars. Because of the narrow genetic base of the cultivars, there is a need to transfer genes of desirable traits from wild to cultivated rice, and it is an essential strategy in pre-breeding. Wild species have beneficial alleles for yield improvement, but the expression of these alleles is frequently masked due to the presence of other detrimental loci. Yet, several yields enhancing quantitative trait loci (QTLs) have been mapped in the last 25 years from wild species of rice for genetical improvement (Swamy and Sarla, 2008; Gaikwad et al., 2021). The wild rice species, *Oryza nivara*, is the closest wild progenitor of cultivated rice *O. sativa* (Haritha et al., 2018). *O. nivara* accessions showed high genetic diversity in its gene pool with adaptability in different environments (Sarla et al., 2003; Juneja et al., 2006) and is a proven choice to improve the yield levels of cultivars (Vaughan et al., 2003, 2008; Swamy et al., 2014; Ma et al., 2016).

An advanced back cross method is a technique to introduce the favorable alleles from wild into cultivar background (Ma et al., 2016). There are many reports on mapping yield QTLs using wild species (Swamy and Sarla, 2008; Swamy et al., 2011, 2014; Wickneswari et al., 2012; Ma et al., 2016; Bhatia et al., 2018). Back cross inbred lines are useful for mapping QTLs and gene pyramiding (Bhatia et al., 2018; Wang et al., 2018). In addition, QTL pyramiding using introgression lines (ILs) is an effective method in molecular breeding for complex traits (Feng et al., 2018). Back cross introgression lines (BILs) derived from cultivar/wild crosses or alien introgression lines were known for improving yield traits along with different desirable traits, such as quality-related traits and biotic and abiotic stress resistance (Mahmoud et al., 2008; Brar and Singh, 2011; Swamy et al., 2012, 2014). BILs generated by repeated backcrosses are useful in restoring the pollen fertility and eliminating the undesirable trait effects on the cultivar background (Swamy and Sarla, 2008). Several studies used BC_2_F_2_ populations derived from interspecific crosses to map QTLs. However, when introgression lines that are genetically similar to each other but phenotypically different are used, e.g., near-isogenic lines, the power to map the phenotype increases considerably. Compared to BC_2_F_2_, these advanced ILs in BC_2_F_8_ do not simultaneously have the confounding effects of segregation of several QTLs all over the genome or the complex epistatic interactions in the genome. Therefore, two high-yielding stable, fine grain BC_2_F_8_ Ils 166s and 14s derived from Swarna/*O. nivara* cross, were chosen to map QTLs for yield-related traits. These BC_2_F_8_ are derived from 166s (IET21938) and 14s (IET2274) which were identified as two fine-grain introgression lines earlier in BC_2_F_2_ (Swamy et al., 2012) and were shown in BC_2_F_6–7_ to be high-yielding in multilocation trials (Haritha et al., 2018) and are also salt tolerant (Ganeshan et al., 2016). The selected parental lines, *viz.*, 166s and 14s with 75.8 and 77.8% recurrent parent genome, respectively, were part of a library of Swarna chromosome segment substitution lines (CSSLs) having chromosome segment substitutions from *O. nivara* (Balakrishnan et al., 2016; Surapaneni et al., 2017) and showed stability in yield levels over the generations. Among several traits to differentiate the two lines, 166s has a larger number of grains, and 14s has higher thousand-grain weight. Both of which are major yield contributing traits (Supplementary Table 1).

Previously, Pang et al. (2017) and Feng et al. (2018) reported QTLs in the population, generated by crossing Ils developed from local rice varieties. Similarly, QTLs were reported in the F_2_ population derived from a cross between a back cross inbred line (*japonica* × *indica*) and a *japonica* cultivar Z550 (Wang et al., 2018). Various studies reported QTLs for yield traits in rice using primary mapping populations like F_2:3_ (Sabouri et al., 2009; Zhang et al., 2010; Kim et al., 2014; Tian et al., 2015; Biswas et al., 2017; Kumar et al., 2019) and F_2:4_ (Rabiei et al., 2015; Verma et al., 2017; Jeon et al., 2018; Kumar et al., 2019). Crossing of two wild-derived back cross inbred lines generate hybrid lines which can closely resemble the parental lines (Wang et al., 2018) with combination of improved traits. We selected these two high yielding BILs with contrasting yield contributing traits to develop F_2:3:4_ populations with the objective of mapping consistent and precise QTLs for yield-related traits across three generations.

## Materials and Methods

### Plant Material and Field Evaluation

**FIGURE 1:**
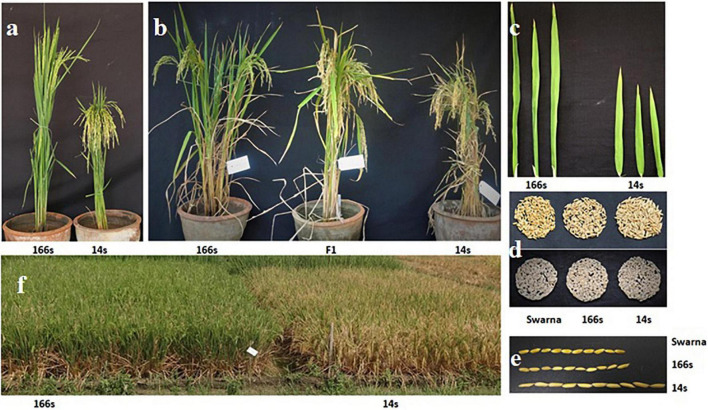
Phenotypic characteristics of parental lines, plant, and grain traits of 166s, 14s, and Swarna. **(a)** Parental lines 166s and 14s, **(b)** 166s, 14s parents with F_1_ (166s × 14s hybrid-middle one) plant, **(c)** Flag leaf length of 166s and 14s, **(d,e)** Swarna, 166s and 14s grains with husk and without husk and length of grains, **(f)** Field view of 166s and 14s.

Initially, a set of back cross introgression lines at BC_2_F_2_ were developed from a cross of Swarna × *O. nivara* (Swamy et al., 2014), and this material was advanced up to BC_2_F_8_ through single panicle selection of selected individual lines. These lines were screened for three consecutive seasons to study G × E interaction of yield and related traits, and two BC_2_F_8_ lines, *viz.*, 166s and 14s, were identified as most stable BILs for different yield-related traits (Balakrishnan et al., 2016; Kavitha et al., 2019). BIL 166s had high biomass, total dry matter, number of grains, seedling vigor, and high photosynthetic rate compared to parent Swarna and was also tolerant to aerobic and saline conditions based on our previous studies. BIL 14s had high single plant yield, bulk yield, per-day productivity, harvest index, 1,000 grain weight, and low unfilled grains. These BC_2_F_8_ BILs, 166s [IET27223] as female parent and 14s [IET 26772] as male parent, were taken as starting material for the experiment and F_1_ was generated by crossing them (Figure 1). In *Rabi* 2015, F_1_ plant was raised and selfed to generate F_2_ mapping population. F_2_ population was raised in *Kharif 2015* and forwarded to F_3_ and F_4_ populations in *Rabi* 2016 and *Rabi* 2018, respectively, at the Indian Institute of Rice Research (IIRR) field. The farm is located in Hyderabad, India at 17° 19′ N latitude and 78° 29′ E longitude.

Field evaluation of yield-related traits in the F_2_ population was carried out in normal irrigated field conditions during *Kharif 2015* using the standard evaluation system of IRRI (SES, IRRI) for 19 phenotypic traits. These were days to initial flowering (DIF), plant height (PH), tiller number (TN), productive tiller number (PTN), flag leaf length (FLL), leaf length (LL), leaf width (LW), culm length (CL), single plant yield (SPY), biomass (BM), total dry matter (TDM), harvest index (HI), panicle length (PL), filled grains (FG), unfilled grains (UFG), grain number (GN), spikelet fertility (SPF), panicle weight (PW), and thousand-grain weight (TGW). In addition, 18 of these traits were also measured in F_3_ population, viz., DIF, PH, TN, PTN, CL, SPY, BM, TDM, HI, PL, FG, UFG, GN, SPF, PW, TGW, PDP, and Bulk yield (BY), and 8 traits in F_4_ population, viz., PH, TN, PTN, SPY, BM, TDM, HI, and TGW, in randomized with three replications.

### DNA Extraction

Fresh leaf samples from 1-month old seedlings were collected from 174 F_2_ plants and parents in *Kharif 2015.* DNA was extracted using cetyltrimethyl ammonium bromide (CTAB) mini prep method (Doyle and Doyle, 1990). In this method, 400 μl of CTAB was added to small pieces of leaf in a mini prep plate for grinding. Four hundred microliters of CTAB was added to it and was mixed as well in a 2 ml microcentrifuge tube. To the leaf sample, equal amounts of chloroform:isoamyl alcohol (24:1) was added in each tube while shaking vigorously until it was dissolved. Then tubes were kept for centrifugation at 15,000 rpm for 15 min, and the supernatant was removed gently into a fresh 1.5 ml microcentrifuge tube. An equal amount of isopropanol was added to this supernatant before it was mixed slowly and kept in a freezer at −20°C for about 10 min to 2 days for DNA pellet formation. Afterward, these tubes were centrifuged at 10,000 rpm for 10 min, and the supernatant was removed in this step very slowly to retain the pellet in the tube. Then, 100 μl of 70% of ethanol was added to the pellet and kept for centrifugation at 8,000 rpm for 5 min for pellet cleaning. This step was repeated again to get a clear pellet of DNA before this DNA pellet was dissolved in 100 μl of 1× TE buffer or distilled water for further use.

### Genotyping

Parental polymorphism between 166s and 14s using 830 simple sequence repeats (SSRs) was conducted. Out of this, only 79 showed polymorphism. F_2_ population of 174 plants was screened using these 79 primers of which only 64 were clearly segregated in the population. The remaining 15 primers only showed 14s kind of bands. Polymerase chain reaction (PCR) was carried out using 10 μl reaction mixture containing 3 μl of DNA sample (50 ng), 3.8 μl of millipore water, 0.1 μl of dNTPs, 1.2 μl of 25 mM Mgcl_2_, 1 μl of 10× PCR buffer with Mg, 0.8 μl of SSR primer, and 0.1 μl of taq polymerase in each PCR plate. PCR was conducted with the initial denaturation step maintained for 5 min at 95°C. It was also conducted for each cycle with a denaturation temperature of 95°C for 30 s, annealing temperature of 55°C for 30 s, and an extension temperature of 72°C for 30 s which was maintained for up to 35 cycles before a final extension of up to 7 min at 72°C. The final PCR product after 35 cycles was maintained at a temperature of 10°C before collecting from PCR. For agarose gel electrophoresis, 3% gels were prepared with wells to load the DNA samples, and electrophoresis was carried out at 180 volts in 0.5× Tris/Borate/EDTA (TBE) buffer. The band pattern was documented in UV-light in the gel documentation unit. Gel scoring of samples along with parents was conducted by noting ‘A’ (P1) for homozygous 166s allele, ‘B’ (P2) for homozygous 14s allele, and ‘H’(P1P2) for heterozygous, i.e., presence of both the alleles.

### Statistical Analyses and Quantitative Trait Loci Mapping

Analysis of variance (ANOVA) was performed using the statistical tool for agricultural research (STAR v2.0.1) software, and association between the traits was estimated with plant breeding tools (PB tools) (Ver. 1.4^1^) using Pearson’s product-moment correlation method at the significant levels of *p = 0.05–0.001 and ^**^p ≥ 0.001. QTL mapping involves the construction of a linkage map and QTL analysis. Inclusive component interval mapping (ICIM) QTL mapping (IciMapping v4.1) integrated software (Wang, 2009^2^) was used for both linkage mapping and QTL analysis. Single marker analysis, interval mapping, and composite interval mapping were performed using this software using F_2_ genotypic data and F_2_, F_3_ (F_2:3_), and F_4_ (F_2:4_) phenotypic data for QTL mapping. Further genotypic dissection within a major QTL region for TGW was carried out in the extreme phenotypes using 5 low TGW (LTGW) and 5 high TGW (HTGW) F_2_ lines with SSR markers within the detected QTL region. The co-segregating markers were then used to genotype 10 LTGW and 10 HGTW F_4_ lines.

## Results

### Phenotyping F_2_, F_3_, and F_4_ Populations

Yield-related traits of populations were evaluated in Kharif (wet season) 2015, Rabi (dry season) 2016, and Rabi (dry season) 2018 for F2, F3, and F4, respectively, using standard evaluation system (SES) of Standard Evaluation System for Rice [SES] (2014). The yield-related traits in F_2_ (19 traits), F_2:3_ (18 traits), and in F_2:4_ (8 traits) were measured and significant differences among the individuals or lines were observed in the three populations for each trait. The SPY of lines C_2_12, C_2_124, C_2_128, C_2_143, and C_2_ 162 showed a positively significant difference with Swarna and P_2_ in F_3_ (Supplementary Tables 2, 3) and showed higher yield in F_3_ and F_4_. Supplementary Table 2 shows the cumulative number of lines that are significantly different than parents for each trait. The details of significantly different traits in individual lines as compared to parents are given in Supplementary Table 3. The descriptive statistical data for all these yield-related traits of F_2_, F_3_, and F_4_ populations is given in Table 1, and frequency distribution is shown in Supplementary Figures 1–3, respectively. Box plots for 8 traits in F_2_, F_3_, and F_4_ and 16 traits in F_2_ and F_3_ are shown in Supplementary Figures 4, 5, respectively.

**TABLE 1:** Descriptive statistics for the yield-related traits in F_2_, F_3_, and F_4_ population of 166s × 14s.

Trait	Generation	Minimum	Maximum	Mean	Median	Range	Variance	Standard Deviation	Critical value	Skewness	Kurtosis
DIF	F_2_	88	135	109.25	108	47	48.24	6.95	6.36	0.43	0.83
	F_3_	88	129	98.87	98	41	42.26	6.5	6.58	1.26	3.01
	F_4_	−	−	−	−	−	−	−	−	−	−
PH	F_2_	50	100	77.04	77	50	87.52	9.36	12.14	0.03	0.11
	F_3_	59.33	96	75.19	75.33	36.67	42.86	6.55	8.71	0.25	0.39
	F_4_	68.67	98.33	84.42	84.00	29.66	38.48	6.20	7.35	0.04	−0.19
TN	F_2_	2	40	18.25	17	38	64.2	8.01	43.91	0.54	−0.25
	F_3_	7.67	28.33	14.46	14	20.66	13.63	3.69	25.52	0.65	0.47
	F_4_	6.33	20.25	12.60	12.33	13.92	6.14	2.48	19.67	0.67	0.60
PTN	F_2_	2	38	16.17	15	36	43.93	6.63	41	0.6	0.34
	F_3_	7.33	26	14.06	13.33	18.67	13.22	3.64	25.86	0.68	0.29
	F_4_	6.33	20.00	12.59	12.33	13.67	6.00	2.45	19.46	0.60	0.39
CL	F_2_	34	72.83	51.7	51	38.83	47.67	6.9	13.35	0.46	0.45
	F_3_	39.33	72	53.41	53	32.67	38.28	6.19	11.59	0.44	0.32
	F_4_	−	−	−	−	−	−	−	−	−	−
SPY	F_2_	1.6	75.1	25.61	23.4	73.5	205.22	14.33	55.93	0.73	0.49
	F_3_	1.8	32.2	16.34	16.9	30.4	38.48	6.2	37.95	0.01	−0.32
	F_4_	6.23	42.73	22.99	22.73	36.50	38.31	6.19	26.92	0.36	0.39
BM	F_2_	2.78	85.8	27.77	25.4	83.02	213.82	14.62	52.66	0.82	0.88
	F_3_	2.4	49.88	13.44	12.1	47.48	44.58	6.68	49.7	1.77	5.56
	F_4_	5.53	52.50	25.68	25.03	46.97	157.79	12.56	48.92	0.19	−1.24
TDM	F_2_	4.38	138.8	53.18	48.4	134.42	745.7	27.31	51.35	0.61	0.11
	F_3_	8.35	68.88	29.81	28.53	60.53	122.43	11.06	37.11	0.75	1.15
	F_4_	20.30	92.47	48.47	47.57	72.17	208.57	14.44	29.79	0.34	−0.37
HI	F_2_	3.89	69.57	47.31	47.82	65.68	74.83	8.65	18.28	−1.21	3.36
	F_3_	13.89	77.51	55.29	57.33	63.62	132.78	11.52	20.84	−0.97	1.2
	F_4_	13.94	78.64	49.98	48.34	64.70	204.40	14.30	28.60	0.04	−0.92
PL	F_2_	15.37	25.98	21.22	21.28	10.61	3.44	1.85	8.74	−0.24	0.39
	F_3_	17.57	24.3	21.16	21.13	6.73	1.89	1.37	6.49	−0.08	−0.27
	F_4_	−	−	−	−	−	−	−	−	−	−
FG	F_2_	20	191.2	108.96	111	171.2	932.7	30.54	28.03	−0.08	0.07
	F_3_	46.33	160	104.82	103.67	113.67	463.17	21.52	20.53	0.07	−0.19
	F_4_	−	−	−	−	−	−	−	−	−	−
UFG	F_2_	2.4	162.4	25.68	21.6	160	321.61	17.93	69.82	3.13	19.08
	F_3_	2	81.67	19.74	15.5	79.67	198.55	14.09	71.4	1.66	3.34
	F_4_	−	−	−	−	−	−	−	−	−	−
GN	F_2_	38.67	254	134.47	130.8	215.33	1325.93	36.41	27.08	0.2	0.19
	F_3_	70.33	192.5	124.38	120	122.17	620.48	24.91	20.03	0.53	−0.14
	F_4_	−	−	−	−	−	−	−	−	−	−
SPF	F_2_	36.06	98.4	81.13	83.06	62.34	104.45	10.22	12.6	−1.36	2.71
	F_3_	37.77	98.2	84.63	87.29	60.43	97.36	9.87	11.66	−1.8	4.31
	F_4_	−	−	−	−	−	−	−	−	−	−
PW	F_2_	0.57	3.45	2.12	2.13	2.88	0.32	0.57	26.73	−0.27	0.21
	F_3_	1.05	3.26	1.92	1.9	2.21	0.15	0.38	20.01	0.36	0.49
	F_4_	−	−	−	−	−	−	−	−	−	−
TGW	F_2_	10.2	23.8	19.44	19.6	13.6	4.61	2.15	11.05	−0.62	1.21
	F_3_	7.9	23.2	17.53	17.3	15.3	4.27	2.07	11.8	−0.25	2.24
	F_4_	14.20	25.33	20.44	20.50	11.13	3.40	1.85	9.03	0.01	0.20
PDP	F_2_	0.01	0.55	0.19	0.17	0.54	0.01	0.11	57.75	0.79	0.56
	F_3_	0.01	0.25	0.13	0.13	0.24	0	0.05	38.16	0.02	−0.32
	F_4_	−	−	−	−	−	−	−	−	−	−
BY	F_2_	−		−	−	−	−	−	−	−	−
	F_3_	0.01	0.25	0.13	0.13	0.615	0.01295	0.1133	30.87	0.3036	0.0525
	F_4_	−	−	−	−	−	−	−	−	−	−
FLL	F_2_	14	39.6	26.89	27	25.6	18.39	4.29	15.95	0.06	0.25
	F_3_	−	−	−	−	−	−	−	−	−	−
	F_4_	−	−	−	−	−	−	−	−	−	−
LL	F_2_	21	47	32.99	32	26	24.26	4.92	14.93	0.33	0.02
	F_3_	−	−	−	−	−	−	−	−	−	−
	F_4_	−	−	−	−	−	−	−	−	−	−
LW	F_2_	0.6	1.6	1.14	1.1	1	0.04	0.2	17.93	−0.29	0.1
	F_3_	−	−	−	−	−	−	−	−	−	−
	F_4_	−	−	−	−	−	−	−	−	−	−

*DIF, days to initial flowering; PH, plant height; TN, tiller number; PTN, productive tiller number; CL, culm length; SPY, single plant yield; BM, biomass; TDM, total dry matter, HI, harvest index; PL, panicle length; FG, filled grains; UFG, unfilled grains; GN, grain number; SPF, spikelet fertility; PW, panicle weight; TGW, thousand-grain weight, PDP, per day productivity; BY, bulk yield; FLL, flag leaf length; LL, leaf length; LW, leaf width.*

### Trait Correlation

Correlation analysis among all yield-related traits in F_2_ and F_3_ was evaluated using Pearson’s product-moment correlation and probability values of *p = 0.05–0.001 as significant and ^**^p ≥ 0.001 as a highly significant correlation between traits. Results are shown in Table 2. Some traits consistently showed significant association in F_2_, F_2:3_, and F_2:4_. The traits which showed highly significant positive correlations in F_2_ were as follows: FLL with PH, CL, SPY, BM, HI, PL, FG, UFG, GN, PW, and PDP; LL with PH, TN, PTN, CL, SPY, BM, TDM, HI, PL, FG, UFG, GN, PW, TGW, PDP, and FLL; and LW with PH, TN, PTN, CL, SPY, BM, TDM, PL, FG, UFG, GN, PW, PDP, FLL, LL, and LW. A highly significant negative association was observed for FLL, LL, and LW with DIF.

**TABLE 2:** Correlation coefficients among yield-related traits of F_2_, F_3_, and F_4_ population of 166s × 14s.

		DIF	PH	TN	PTN	CL	SPY	BM	TDM	HI	PL	FG	UFG	GN	SPF	PW	TGW	PDP	FLL	LL	LW
F_2_	DIF	1**																			
F_3_	DIF	1**																			
F_2_	PH	−0.32**																			
F_3_	PH	0.18**																			
F_4_	PH	–	1**																		
F_2_	TN	−0.18**	*0.29* **																		
F_3_	TN	0.01	*0.23* **																		
F_4_	TN	–	–0.02	1**																	
F_2_	PTN	−0.29**	*0.32* **	*0.92* **																	
F_3_	PTN	0.02	*0.24* **	*0.98* **																	
F_4_	PTN	–	–0.01	*0.98* **	1**																
F_2_	CL	−0.21**	*0.80* **	*0.22* **	*0.26* **																
F_3_	CL	0.19**	*0.91* **	*0.26* **	*0.25* **																
F_2_	SPY	−0.33**	*0.47* **	*0.74* **	*0.79* **	*0.36* **															
F_3_	SPY	0.07	*0.41* **	*0.26* **	*0.28* **	*0.42* **															
F_4_	SPY	–	*0.28* **	*0.39* **	*0.39* **	–	1**														
F_2_	BM	−0.27**	*0.53* **	*0.76* **	*0.76* **	*0.43* **	*0.78* **														
F_3_	BM	0.38**	*0.35* **	*0.25* **	*0.24* **	*0.39* **	*0.47* **														
F_4_	BM	–	0.02	*0.19* **	*0.18* **	–	0.08	1**													
F_2_	TDM	−0.31**	*0.52* **	*0.80* **	*0.82* **	*0.40* **	*0.94* **	*0.94* **													
F_3_	TDM	*0.28* **	*0.44* **	*0.30* **	*0.30* **	*0.48* **	*0.84* **	*0.87* **													
F_4_	TDM	–	0.12*	*0.34* **	*0.32* **	–	*0.51* **	*0.89* **	1**												
F_2_	HI	−−0.20**	0.10*	0.09	0.19**	0.06	*0.46* **	–0.09	0.19**												
F_3_	HI	−−0.31**	0.07	–0.03	0	0.05	*0.40* **	−0.52**	–0.09												
F_4_	HI	–	0.11*	0.00	0.02	–	*0.35* **	*−0 .87* **	*−0 .60* **	1**											
F_2_	PL	−0.42**	*0.75* **	0.27**	0.30**	*0.60* **	0.49**	0.47**	0.50**	0.19**											
F_3_	PL	–0.06	*0.29* **	–0.09	–0.08	*0.23* **	0.11*	0.12*	0.13*	–0.02											
F_2_	FG	−0.28**	*0.62* **	0.38**	0.43**	0.50**	0.60**	0.53**	0.59**	0.27**	*0.72* **										
F_3_	FG	0	*0.20* **	–0.05	–0.06	0.16*	0.11*	0.11*	0.13*	0.04	*0.37* **										
F_2_	UFG	0	0.35**	0.02	–0.01	0.27**	–0.01	0.16*	0.08	−−0.27**	0.33**	0.07									
F_3_	UFG	0.20**	0.06	−0.10*	−0.10*	–0.01	–0.07	0.14*	0.05	−−0.19**	0.08	–0.02									
F_2_	GN	−0.22**	*0.69* **	0.33**	0.36**	0.54**	0.50**	0.52**	0.54**	0.09	*0.77* **	*0.87* **	*0.55* **								
F_3_	GN	0.12*	*0.19* **	−0.10*	−0.10*	0.11*	0.05	0.15*	0.12*	–0.06	*0.36* **	*0.84* **	*0.53* **								
F_2_	SPF	−−0.18**	–0.06	0.17**	0.21**	–0.07	0.28**	0.10*	0.20**	*0.41* **	0.05	*0.39* **	−−0.82**	–0.08							
F_3_	SPF	−−0.18**	0	0.07	0.07	0.05	0.09	−0.12*	–0.03	*0.21* **	0.03	*0.30* **	−−0.94**	−0.26**							
F_2_	PW	−0.39**	*0.69* **	0.36**	0.41**	*0.55* **	0.61**	0.53**	0.59**	0.31**	*0.80* **	*0.90* **	0.12*	*0.81* **	*0.33* **						
F_3_	PW	0.02	*0.22* **	−0.10*	−0.10*	*0.17* **	0.14*	0.11*	0.14*	0.08	*0.36* **	*0.72* **	0	*0.61* **	*0.21* **						
F_2_	TGW	−−0.31**	*0.25* **	0.08	0.14*	*0.17* **	*0.24* **	*0.18* **	*0.23* **	*0.22* **	*0.25* **	0.15*	−−0.11*	0.07	*0.23* **	*0.31* **					
F_3_	TGW	0.01	0.06	0.06	0.07	0.08	0.14*	0.05	0.10*	0.07	–0.07	−0.14*	−−0.14*	−−0.20**	0.09	0					
F_4_	TGW	–	*0.18* **	–0.06	–0.03	–	0.12*	–0.08	0.00	0.10*	–	–	–	–	–	–	1**				
F_2_	PDP	−−0.40**	*0.47* **	*0.72* **	*0.78* **	*0.36* **	*1.00* **	*0.77* **	*0.94* **	*0.46* **	*0.50* **	*0.59* **	–0.01	*0.49* **	*0.28* **	*0.61* **	*0.25* **				
F_3_	PDP	–0.07	*0.39* **	*0.27* **	*0.28* **	*0.39* **	*0.99* **	*0.42* **	*0.80* **	*0.44* **	0.12*	0.11*	−0.10*	0.04	0.11*	0.14*	0.13*				
F_3_	BY	0.10*	*0.25* **	0.01	–0.01	*0.29* **	*0.30* **	*0.16* **	*0.27* **	*0.19* **	0.09	0.06	–0.05	0.01	0.06	0.08	0.07	*0.28* **			
F_2_	FLL	−−0.39**	*0.64* **	0.07	0.15*	*0.51* **	*0.32* **	*0.30* **	0.31*	*0.18* **	*0.55* **	*0.50* **	*0.29* **	*0.55* **	–0.04	*0.53* **	0.12*	*0.33* **	1**		
F_2_	LL	−−0.34**	*0.66* **	*0.17* **	*0.24* **	*0.57* **	*0.41* **	*0.42* **	*0.42* **	*0.18* **	*0.58* **	*0.55* **	*0.27* **	*0.58* **	0.01	*0.58* **	*0.22* **	*0.42* **	*0.72* **	1**	
F_2_	LW	−−0.20**	*0.63* **	*0.29* **	*0.23* **	*0.46* **	*0.43* **	*0.48* **	*0.48* **	0.10*	*0.51* **	*0.50* **	*0.27* **	*0.56* **	0	*0.54* **	0.16*	*0.43* **	*0.51* **	*0.60* **	1**

*P = 0.05–0.001, significant lines.*

**P ≥ 0.001, highly significant lines.

*Highly significant positive values in italics, highly significant negative values in bold.*

*F_2_, F_3_, and F_4_-Generations.*

*DIF, days to initial flowering; PH, plant height; TN, tiller number; PTN, productive tiller number; CL, culm length; SPY, single plant yield; BM, biomass; TDM, total dry matter, HI, harvest index; PL, panicle length; FG, filled grains; UFG, unfilled grains; GN, grain number; SPF, spikelet fertility; PW, panicle weight; TGW, thousand-grain weight, PDP, per day productivity; BY, bulk yield; FLL, flag leaf length; LL, leaf length; LW, leaf width. “−” Observations not taken for one of correlated trait.*

In F_3_, highly significant positive correlations were observed for BY with PH, CL, SPY, BM, TDM, HI, and PDP. PH, TN, PTN, CL, BM, TDM, HI, and PDP showed significant and positive association to single plant yield, and significant negative association was observed for TGW, SPF, and HI with UFG in both F_2_ and F_2:3_. In F_2:4_, a highly significant positive association was observed between PTN and TN; SPY with PH, TN, and PTN; BM and TDM with both TN and PTN; TDM with SPY and BM; HI with SPY; and TGW with PH. There was also a highly significant negative association of HI with BM and TDM. A significant positive association was identified for PH with TDM and HI and TGW with SPY.

Traits PH, TN, PTN, TDM, and HI showed a highly significant positive association with single plant yield in all three generations of F_2_, F_3_, and F_4_. TGW showed a highly significant association with SPY in F_2_, and a significant association was observed between TGW and SPY in F_3_ and F_4_. The traits PH, TN, PTN, and TDM consistently showed a highly significant positive association to single plant yield in all three generations. Highly significant positive correlations were observed for the traits SPY with PH, TN, PTN, CL, BM, TDM, HI, and PDP in F_2_ and F_2:3_. The traits which showed a highly significant negative correlation in F_2_ and F_2:3_ were HI with DIF and UFG; SPF with DIF and UFG. A significant negative association between TGW with UFG was also observed, in addition to the highly significant negative association in both F_2:3_ and F_2:4_ between traits HI with BM. A significant positive association was observed between traits TGW with SPY.

### Genotyping

The F_2_ population consisting of 174 lines was genotyped using 79 polymorphic markers. Among these polymorphic SSR markers, segregation distortion was observed for nearly 20 markers. In this study, heterozygous bands ranged from 14.3 (RM1189) to 69.5% (RM430) in the whole population. The 166s (female P1) parental type bands ranged between 1.1% (RM202) and 47.1% (RM1189), while the 14s (male P2) parent type bands ranged from 9.7 (RM430) to 89% (RM3708). The maximum percentage of missing/null alleles was observed for the marker RM4996 (35.6%), followed by RM16649 with 28.1%, and RM1189 with 25.8%. The percentage alleles similar to 166s ranged from 1.15 to 47.13% with an average of 20.89%, while allelic similarity to 14s in the population was 9.77 to 58.62% with an average of 25.74%. Heterozygous bands in the F_2_ population ranged from 14.37 to 69.54% with an average of 43.28%, and non-amplified/missing/null alleles were 0.57–35.63% (Supplementary Table 4). The parental lines of the mapping population were part of the set of CSSLs. 166s had 12.1% and 14s had 17.2% chromosomal segments from *O. nivara* based on genotyping using 111 SSR markers and 75.8 and 77.8% of Swarna alleles, respectively, with remaining heterozygous or unamplified bands. These BC_2_F_8_ parental lines are fixed sib lines, which explain the similarity between them.

### Quantitative Trait Loci Mapping

**FIGURE 2:**
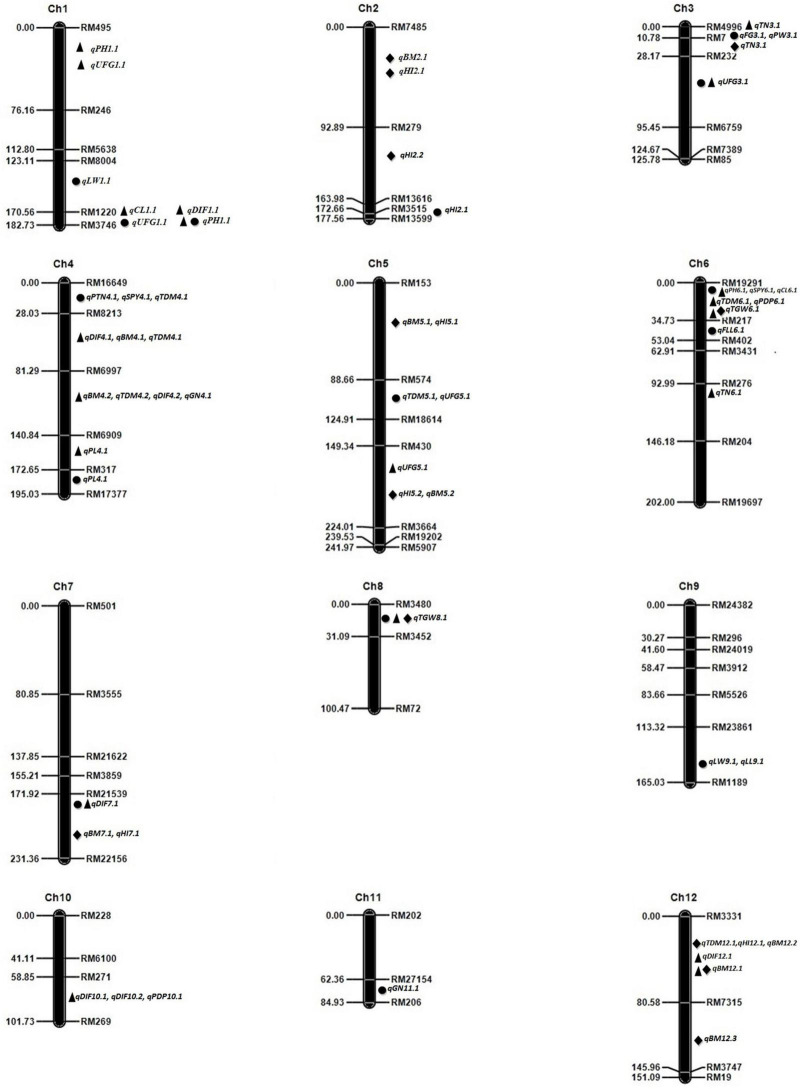
Molecular linkage map of 12 chromosomes with position of Quantitative Train Loci (QTLs) for yield-related traits in F_2:3:4_ generations detected using ICIM. QTLs named by trait abbreviation and chromosome number, ● – F_2_ generation, ▲ – F_3_ generation, ◆ – F_4_ generation. DIF, days to initial flowering; PH, plant height; PTN, productive tiller number; SPY, single plant yield; TDM, total dry matter; HI, harvest index; PL, panicle length; FG, filled grains; UFG, unfilled grains; GN, grain number; PW, panicle weight; TGW, thousand-grain weight; PDP, per day productivity; BY, bulk yield; FLL, flag leaf length; LL, leaf length; LW, leaf width.

F_2_ linkage map was constructed using ICIM v4.1 software. There were 33 QTLs mapped using single marker analysis in F_2_, F_2:3_, and F_2:4_ (Table 3). Eleven QTLs were mapped in F_2_ with phenotypic variance (PV) between 5.17 and 9.71% with a maximum logarithm of odds (LOD) value of 5.04. Thousand-grain weight QTL showed the maximum LOD. In F_2:3_, 17 QTLs were identified with PV between 4.37 and 12.49% with a maximum LOD value of 3.85. Four QTLs were mapped in F_2:4_ with PV from 6.52 to 14.38%. The maximum phenotypic variance explained (PVE) was shown by QTL for thousand-grain weight or plant height in each generation. In F_2_, using inclusive composite interval, mapping 21 QTLs were identified on chromosomes 1, 2, 3, 4, 5, 6, 7, 8, 9, and 11 for 19 yield-related traits, with PV ranging from 4.03 to 12.54%. In F_2:3_, 30 QTLs were identified on chromosomes 1, 3, 4, 5, 6, 7, 8, 10, and 12 for 18 yield-related traits, with PV ranging from 1.32 to 13.01%. In F_2:4_, 17 QTLs were identified on chromosomes 2, 3, 5, 6, 7, 8, and 12 for 8 yield-related traits, with PV ranging from 2.28 to 15.19%. In F_2_, F_2:3_, and F_2:4_ (Figure 2) PV of QTLs ranged from 1.32 to 15.19%.

**TABLE 3:** Additive and dominance effects of quantitative trait loci (QTLs) identified for yield-related traits in F_2_, F_2:3_, and F_2:4_ populations of 166s × 14s using ICIM.

SL. No.	QTL	Chromosome	Generation	Left marker	Right marker	LOD	PVE (%)	Additive	Dominance
1	*qLW1.1*	1	F_2_	RM8004	RM1220	3.15	6.06	0.11	−0.04
2	*qPH1.1*	1	F_2_	RM1220	RM3746	2.54	8.18	2.96	−1.92
3	*qUFG1.1*	1	F_2_	RM1220	RM3746	3.59	2.78	5.92	−5.91
4	*qDIF1.1*	1	F_3_	RM1220	RM3746	4.2	1.94	2.58	0.38
5	*qPH1.1*	1	F_3_	RM495	RM246	3.51	13.01	4.64	−2.45
6	*qPH1.2*	1	F_3_	RM1220	RM3746	4.69	6.63	2.7	0.08
7	*qCL1.1*	1	F_3_	RM1220	RM3746	2.72	8.93	1.73	1.21
8	*qUFG1.1*	1	F_3_	RM495	RM246	8.8	1.63	20.41	−22.33
9	*qHI2.1*	2	F_2_	RM3515	RM13599	3.71	4.12	7.27	8.92
10	*qBM2.1*	2	F_4_	RM7485	RM279	7.14	2.93	−0.7	20.59
11	*qHI2.1*	2	F_4_	RM7485	RM279	3.24	3.62	−0.47	−22.25
12	*qHI2.2*	2	F_4_	RM279	RM13616	2.84	3.52	−1.59	−21.53
13	*qFG3.1*	3	F_2_	RM4996	RM7	2.53	9.65	−8.87	11.28
14	*qPW3.1*	3	F_2_	RM4996	RM7	3.05	9.06	−0.19	0.21
15	*qUFG3.1*	3	F_2_	RM232	RM6759	10.04	8.26	4.25	−19.43
16	*qTN3.1*	3	F_3_	RM4996	RM7	3	8.35	−0.25	1.75
17	*qUFG3.1*	3	F_3_	RM232	RM6759	5.19	1.71	13.53	−14.82
18	*qTN3.1*	3	F_4_	RM7	RM232	2.53	6.56	−0.9	1.22
19	*qPL4.1*	4	F_2_	RM317	RM17377	3.16	8.17	0.54	0.62
20	*qPTN4.1*	4	F_2_	RM16649	RM8213	2.52	5.2	−3.75	−1.65
21	*qSPY4.1*	4	F_2_	RM16649	RM8213	2.6	8.5	−7.26	−4.34
22	*qTDM4.1*	4	F_2_	RM16649	RM8213	2.65	8.97	−14.53	−7.29
23	*qDIF4.1*	4	F_3_	RM8213	RM6997	2.67	5.06	−3.83	−3.41
24	*qDIF4.2*	4	F_3_	RM6997	RM6909	4.47	6.98	−4.82	−4.19
25	*qBM4.1*	4	F_3_	RM8213	RM6997	7.68	4.29	−4.25	−4.95
26	*qBM4.2*	4	F_3_	RM6997	RM6909	6.41	4.22	−4.24	−5.17
27	*qTDM4.1*	4	F_3_	RM8213	RM6997	5	8.54	−6.84	−7.01
28	*qTDM4.2*	4	F_3_	RM6997	RM6909	4.1	8.45	−7.03	−6.72
29	*qPL4.1*	4	F_3_	RM6909	RM317	2.56	6.74	0.46	0.09
30	*qGN4.1*	4	F_3_	RM6997	RM6909	3.59	3.63	15.35	−27.04
31	*qTDM5.1*	5	F_2_	RM574	RM18614	2.61	4.03	−11.01	8.36
32	*qUFG5.1*	5	F_2_	RM574	RM18614	3.39	5.18	10.04	−9.86
33	*qUFG5.1*	5	F_3_	RM430	RM3664	5.25	1.83	−13.16	−13.73
34	*qBM5.1*	5	F_4_	RM153	RM574	10.15	3.23	−4.47	−22.17
35	*qBM5.2*	5	F_4_	RM430	RM3664	8.27	2.94	−1.43	20.49
36	*qHI5.1*	5	F_4_	RM153	RM574	4	3.76	2.22	22.88
37	*qHI5.2*	5	F_4_	RM430	RM3664	4.24	3.69	2.35	−22.06
38	*qFLL6.1*	6	F_2_	RM217	RM402	2.87	7.5	1.57	0.73
39	*qPH6.1*	6	F_2_	RM19291	RM217	3.75	12.54	−1.46	−4.93
40	*qSPY6.1*	6	F_2_	RM19291	RM217	2.65	6.71	−3.94	−7.16
41	*qPH6.1*	6	F_3_	RM19291	RM217	4.52	7.07	−2.34	−1.38
42	*qTN6.1*	6	F_3_	RM276	RM204	3.59	10.08	0.11	−1.91
43	*qCL6.1*	6	F_3_	RM19291	RM217	3.09	9.81	−1.48	−1.55
44	*qSPY6.1*	6	F_3_	RM19291	RM217	2.95	6.51	−0.82	−3.93
45	*qTDM6.1*	6	F_3_	RM19291	RM217	2.86	2.36	−1.59	−5.29
46	*qTGW6.1*	6	F_3_	RM19291	RM217	2.51	9.33	0.78	−1.54
47	*qPDP6.1*	6	F_3_	RM19291	RM217	3.06	6.39	−0.01	−0.04
48	*qTGW6.1*	6	F_4_	RM19291	RM217	3.52	15.19	0.56	−2.06
49	*qDIF7.1*	7	F_2_	RM21539	RM22156	3.22	8.48	2.81	0.45
50	*qDIF7.1*	7	F_3_	RM21539	RM22156	4.84	8.65	5.47	−3.89
51	*qBM7.1*	7	F_4_	RM21539	RM22156	6.51	2.92	1.36	20.48
52	*qHI7.1*	7	F_4_	RM21539	RM22156	3.39	3.55	−1.91	−21.6
53	*qTGW8.1*	8	F_2_	RM3480	RM3452	4.27	9.86	−1.53	0.82
54	*qTGW8.1*	8	F_3_	RM3480	RM3452	5.43	7.48	−1.21	0.03
55	*qTGW8.1*	8	F_4_	RM3480	RM3452	5.87	6.11	−0.98	−0.08
56	*qLL9.1*	9	F_2_	RM23861	RM1189	2.65	6.91	0.87	−3.91
57	*qLW9.1*	9	F_2_	RM23861	RM1189	3.14	8.9	0.06	−0.15
58	*qDIF10.1*	10	F_3_	RM271	RM269	4.41	2	2.52	−0.44
59	*qDIF10.2*	10	F_3_	RM271	RM269	2.71	1.32	−1.39	2.42
60	*qPDP10.1*	10	F_3_	RM271	RM269	2.82	7.1	0.03	0.02
61	*qGN11.1*	11	F_2_	RM27154	RM206	2.57	5.61	19.7	−15.02
62	*qDIF12.1*	12	F_3_	RM3331	RM7315	2.77	7.64	4.76	−5.87
63	*qBM12.1*	12	F_3_	RM3331	RM7315	3.96	4.09	4.4	−5.52
64	*qBM12.1*	12	F_4_	RM3331	RM7315	6.18	2.97	2.4	−20.75
65	*qBM12.1*	12	F_4_	RM3331	RM7315	7.63	2.98	−2.27	20.87
66	*qBM12.2*	12	F_4_	RM7315	RM3747	5.4	2.92	−2.26	20.3
67	*qTDM12.1*	12	F_4_	RM3331	RM7315	2.72	2.28	4.29	−21.8
68	*qHI12.1*	12	F_4_	RM3331	RM7315	3.38	3.6	0.59	−22.19

*QTLs named by trait abbreviation and chromosome number, LOD, logarithm of odds; PVE%, phenotypic variance explained by the QTL.*

*DIF, days to initial flowering; PH, plant height; PTN, productive tiller number; SPY, single plant yield; TDM, total dry matter; HI, harvest index; PL, panicle length; FG, filled grains; UFG, unfilled grains; GN, grain number; PW, panicle weight; TGW, thousand-grain weight; PDP, per day productivity; BY, bulk yield; FLL, flag leaf length; LL, leaf length; LW, leaf width. −ve values indicate additive and dominance effect from male parent (14s).*

*Consistent QTL in 3 generations are shown in bold.*

*>20 dominance values was observed with 14s allele for 10 QTLs (5 QTLs for HI, 2 QTLs for BM, 1 QTL each for TDM, UFG, and GN).*

*>20 dominance values was observed with166s allele for 5 QTLs (1 QTL for HI and 4 QTLs for BM).*

### Pleiotropic Region for Quantitative Trait Loci

Many QTLs were clustered in the same region. There were six such regions found on chromosomes 1, 3, 4, 5, 6, and 9 in F_2_ mapping population, on chromosome 1, 4, 6, 10, and 12 in F_2:3_ mapping, and on chromosome 2, 5, 7, and 12 in F_2:4_ mapping population. In F_2_, three loci with the same marker interval responsible for multiple traits of yield QTLs were *qPTN4.1*, *qSPY4.1*, and *qTDM4.1* for productive tiller number, single plant yield, total dry matter mapped at RM16649- RM8213 on chromosome 4, and two QTLs *qPH1.1*, *qUFG1.1* identified in the region between RM1220 and RM3746. Two QTLs for filled grain *qFG3.1* and panicle weight *qPW3.1* mapped between RM4996 and RM7, and two QTLs *qUFG5.1* and *qTDM5.1* mapped at interval RM574- RM18614. *qPH6.1* and *qSPY6.1* were also identified between the same locus between RM19291 and RM217 on chromosome 6. Two QTLs, *qLL9.1* and *qLW9.1*, for leaf length and leaf width were located between RM23861 and RM1189 on chromosome 9.

In F_2:3_, the QTLs for *qPH6.1, qCL6.1, qSPY6.1, qTDM6.1, qTGW6.1*, and *qPDP6.1 are* located at RM19291-RM217 marker interval were detected. Four QTLs, *qDIF4.2*, *qBM4.2*, *qTDM4.2*, and *qGN4.1*, were located between RM6997 and RM6909. Three QTLs, *qDIF1.1, qPH1.2*, and *qCL1.1*, were located in the same marker interval RM1220 - RM3746. QTLs *qDIF4.1*, *qBM4.1*, and *qTDM4.1* were located in the same locus between RM8213 and RM6997, while the locus between RM271 and RM269 harbored *qDIF10.1, qDIF10.2*, and *qPDP10.1* QTLs. Two QTLs for *qPH1.1* and *qUFG1.1* between RM495-RM246 and *qBM12.1* and *qDIF12.1* were located in the same marker interval of RM3331-RM7315.

In F_2:4_, the QTLs for biomass and harvest index were colocated at marker interval regions of RM7485-RM279, RM153-RM574, and RM21539-RM22156, RM3331-RM7315 on chromosomes 2, 5, 5, 7, and 12, respectively. Harvest index (HI) QTL was associated with the last cluster on chromosome 12. The regions strongly associated with more than one trait in both F_2_, F_2:3_ include the region between RM19291 and RM217 which showed a cluster of QTLs for PH and SPY in F_2_ and PH, CL, SPY, TDM, TGW, and PDP in F_3_. The other region, RM1220-RM3746, showed a cluster of QTLs for PH and UFG in F_2_ and DIF, PH, and CL in F_2:3_.

### Consistent Quantitative Trait Loci Across Generations

**FIGURE 3:**
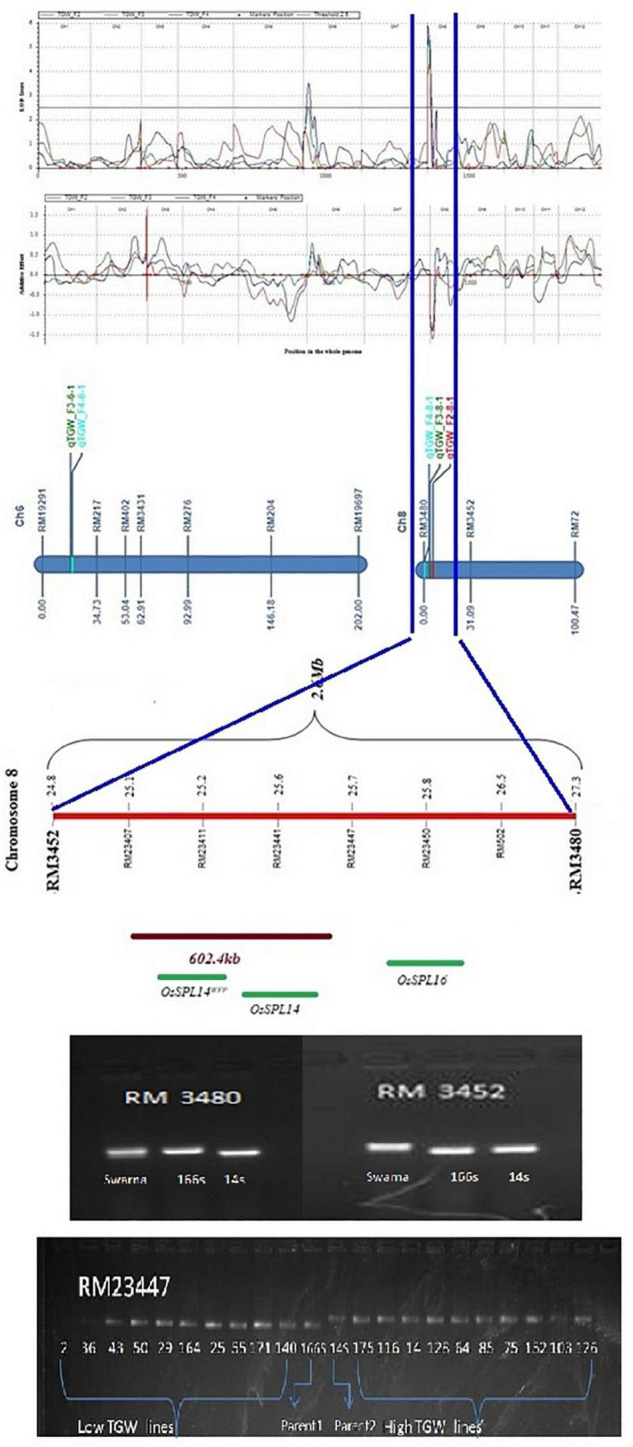
Genetic dissection of *qTGW8.1* QTL from 2.6 Mb region between RM3480-RM3452 and 602.4 Kb region between RM23407 and RM23447 based on genotypic segregation in high and low TGW phenotypes in F_4_.

In all three populations, the QTL *qTGW8.1* for thousand-grain weight was consistently mapped at the same marker interval RM3480- RM3452 on chromosome 8 with LOD ranging from 4.2 to 5.8 and PV ranging from 6.1 to 9.8% (Figure 3). QTLs detected in only F_2_ and F_2:3_ were for PH and SPY between RM19291 and RM217 on chromosome 6. Three QTLs, *qDIF7.1, qBM12.1*, and *qTGW6.1*, were detected in only F_2:3_ and F_2:4_. *qTN3.1* was mapped in F_2:3_ and F_2:4_, but with different marker intervals. QTL *qTGW8.1* was selected for further genomic dissection as it was consistently identified in F_2_, F_3_, and F_4_ generations at the same 2.6 Mb region. SSR markers located between these flanking markers were selected to enrich this 2.6 Mb region and used for further genotyping. Five lines each for high and low TGW were selected in F_2_ based on mean TGW and if the same high or low TGW trait was present in at least two of the three generations. First, F_2_ leaf DNA samples of five lines, each with high and low TGW, were separately used for genotyping with eight SSR markers within QTL. These samples showed clear polymorphism between high and low TGW lines. Marker trait association was detected between TGW and the locus RM 502- RM3480 with a PVE of 47% at LOD 1.49. Later, DNA samples of 10 high and 10 low TGW F_4_ lines were taken and genotyped using two co-segregating markers for validation (Figure 3). The high (20.1 to 25.3 g) and low (15.2 to 18.1 g) TGW lines were significantly different from both parents 166s (17.2 g) and 14s (21.6 g) as well as from Swarna (14.2 g). Genotyping of contrasting lines with 8 SSRs showed that two markers RM23407 and RM23447 showed clear polymorphism between high and low TGW lines in F_2_. However, only RM23447 showed polymorphism between extreme TGW phenotypes of F_4_. In F_2_, among five low TGW lines, the percentage of 166s type alleles was higher than 14s alleles, while the five high TGW lines showed more of 14s type alleles than 166s type. Considering all alleles of the 5 high TGW lines, 50% alleles were of 14s type in C2 117, and 37.5% alleles were of 14s type in the other four lines (C2 103, C2 126, C2 128, and C2 152). Three lines (C2 103, C2 126, and C2 128) had 25% heterozygous alleles and two lines C2 117, and C2 152 had only 12.5% heterozygous alleles. The statistically significant lines identified in our study, compared to both the parents and their common cultivar parent Swarna, were further used for small-scale fine mapping of *qTGW8.1* to detect causative genes for grain weight improvement (Figure 3). Five lines, each with high and low TGW, were selected in F_2_ based on their extreme placement in the frequency distribution curve and if the same high or low TGW trait were present in the same extremes. Likewise, at least two of the three generations were genotyped using SSR markers within the QTL region. Trait marker association using interval mapping detected RM502-RM3480 with PVE of 47% at 1.49 LOD, but no trait association was detected with RM23447 even though 2 groups showed clear co-segregation. Further genotyping in F_4_ identified two co-segregating markers, RM23407 and RM23447, covering a 602.4 kbp region {25148785bp to 25751225bp [QTARO database (affrc.go.jp)]} within *qTGW8.1*.

### Quantitative Trait Loci Clusters Detected

In this study, among all QTLs detected in F2, F2:3, and F2:4, two specific marker regions were strongly associated with more than one trait. RM19291-RM217 had a cluster of QTLs for PH, SPY in F2, and PH, CL, SPY, TDM, TGW, and PDP in F_3_. The other region of RM1220- RM3746 had a cluster of PH, UFG in F_2_, and DIF, PH, and CL in F_2:3_. In addition, the QTLs present in each region were correlated except PH with UFG in F2:3 mapping. In F_2:4_, one specific marker interval, RM3331- RM7315, had a cluster of QTLs for BM (two QTLs), TDM, and HI on chromosome 12. In addition, the region at marker intervals of RM153- RM574 and RM430- RM3664 had a cluster of QTLs for BM and HI on chromosome 5. Lastly, the same QTL cluster for BM and HI were identified on chromosomes 2 and 7 at the regions RM7485-RM379 and RM21539-RM22156, respectively.

## Discussion

Phenotyping the mapping population for 3 generations showed a significant positive association of yield contributing traits PH, TN, PTN, and TDM to single plant yield. Consistent character association revealed the importance of tiller number per plant, productive tillers per plant, and thousand-grain weight as selection criteria for effective yield improvement. Our study showed that F_2_ population is more powerful for detecting QTLs of additive effect and can also be used to estimate the degree of dominance for detected QTLs. All the QTLs mapped in F_4_ were observed with low additive effects from both the parent alleles. A high dominant effect from male parent alleles for the 9 QTLs *qBM5.1*, *qBM12.1*, *qTDM12.1*, *qHI2.1*, *qHI2.2*, *qHI5.2*, *qHI7.1*, *qHI2.1*, and *qTGW6.1* was also observed. Dominance effects were high for the QTLs mainly because of the superiority of heterotic loci identified with low phenotypic variances. QTLs with the low dominant effects showed high phenotypic variances except for the QTLs FG and UFG in F_2_. About 38.1% in F_2_ and 30% in F_2:3_ QTLs explained positive dominance effect, and 61.9% QTLs in F_2_ and 70% in F_2:3_ explained negative dominance values. The QTL *qTGW6.1* showed additive effects from female parents in F_3_ (0.78) and F_4_ (0.56), and also showed dominance effects from male parents in both F_3_ (−1.54) and F_4_ (−2.06). *qTGW8.1* showed a higher additive effect (*O. nivara*) in F_2_ than in F_3_ and F_4_, along with a small dominance effect (0.82) in F_2_. The high dominance effects were identified for the QTLs in F_4_ (82%), followed by F_2_ (14.2%), and F_3_ (13.2%).

In this study, significant QTLs were identified in F_2_, F_2:3_, and F_2:4_, with 9.0–15.19% PV for several yield-related traits. Trait enhancing allele in major QTLs *qPH1.1*, *qTGW6.1*, and *qTGW8.1* and minor QTLs *qGN4.1* and *qTDM5.1* was from *O. nivara*. Swamy et al. (2011, 2012) identified 40% of *O. nivara* alleles were trait enhancing in QTLs in Swarna/*O. nivara* BC_2_F_2_ populations. Sarla (2014) reported that two QTLs *yld9.1* (yield) and *nfg9.1* (number of filled grains) were from *O. nivara* in BIL 248S (DRRDhan40), derived from the same cross. Surapaneni et al. (2017) identified 15 QTLs in a 94 BILs mapping population using 111 SSRs and, of these, 26% QTLs had trait enhancing alleles from *O. nivara*. For the yield QTLs *qSPY4.1* and *qSPY6.1*, trait enhancing allele was from Swarna. The common QTLs *qPH1.1* and *qTGW8.1* at RM1220-RM3746 and RM3480-RM3452, respectively, in F_2_ and F_2:3_ shared the same locus. In these QTLs, PV value was reduced (*qPH1.1*-6.63% and *qTGW8.1*-7.48%) and LOD values increased (*qPH1.1*-4.69 and *qTGW8.1*-5.43) in F_2:3_ compared to F_2_. This reduction of the phenotypic variance of the traits in advanced generations may be due to a reduction in heterozygosity and stabilization of introgression lines. Another common QTL for single plant yield *qSPY6.1* at the locus between RM19291 and RM217 in F_2_ and F_2:3_ showed no significant change in LOD (2.65 in F_2_, 2.95 in F_3_) and PV (6.71% in F_2_, 6.51% in F_3_) values across the generations.

### Major Yield Quantitative Trait Loci Identified in This Study

Among the eight major QTLs, six *qPH1.1, qPH6.1*, *qTGW6.1*, *qFG3.1*, *qTN6.1*, and *qCL6.1* were previously reported in the population derived from cultivated rice varieties for the same trait. The other 2 QTLs, *qTGW8.1* and *qPW3.1*, were reported in wild introgression lines in different genomic regions. *qPH1.1* was identified with the same flanking marker, RM246, by Zhang et al. (2010). *qPH1.1* was also reported previously from *O. nivara* (Swamy et al., 2014; Surapaneni et al., 2017; Haritha et al., 2018; Balakrishnan et al., 2020). *qTGW6.1* was reported previously by Zhu et al. (2019) in the *O. sativa* recombinant inbred line (RIL) population [Teqing/IRBB lines (TI), Zhenshan97/Milyang46 (ZM) and Xieqingzao/Milyang46 (XM)] within the marker region of our study (Table 4).

**TABLE 4:** Major QTLs identified in present study compared to previously reported QTLs.

S. No.	Major QTLs identified in present study	Flanking markers	Position (Mb)	Previous reports of QTLs/genes in this region	Parental lines	References
1	*qTGW8.1*	RM3480-RM3452	2.614	*gw8.1* (RM531-RM42)	GW QTL region from *O. rufipogon* wild F101 (BIL)/Hwaseongbyeo	Xie et al., 2006
				*gw8.1* (RM23201-RM23208)	GW QTL from *O. rufipogon* wild NIL/NIL	Kang et al., 2018
				*qTGW8* (RM6845)	Nipponbare/Z550	Wang et al., 2016
				*qTGW8* (SNPs)	PA64s/CSSLs and CSSL/9311	Lin et al., 2020
2	*qTGW6.1*	RM19291-RM217	3.01	*qTGW6.1* (C358)	*japonica* (Nipponbare)/*indica* (Kasalath)	Ishimaru, 2003
				*qTGW6.1* (RM276-RM136)	ZGX1/IR75862 IL population	Zhang et al., 2018
				*qTGW6.1* (RM402-RM5963)	F_2:3_ developed from *indica* rice lines	Sun et al., 2019
				*qTGW6* (RM589-190)	Teqing/IRBB lines (TI), Zhenshan 97/Milyang 46 (ZM) and Xieqingzao/Milyang 46 (XM) RIL population	Zhu et al., 2019
3	*qPH1.1*	RM495-RM246	27.1	*qph1.1* (RM246)	PA64s (*indica*)/Nipponbare (*japonica*)	Zhang et al., 2010
				*qPH1* (RM237-RM246)	Sepidrood/Gharib Indica varieties	Rabiei et al., 2015
				*qPH1-1* (RM128)	CSSL population (9311/*O. rufipogon*)	Qiao et al., 2016
				*qPH1.1* (RM226-RM431)	Swarna/*O. nivara* BIL population	Surapaneni et al., 2017
						Haritha et al., 2018
4	*qPH6.1*	RM19291-RM217	3.01	*qPH-6* (RM162-RM412)	Xiaobaijingzi/Kongyu 131 RIL	Xing et al., 2014
				*qph6.1* (N6255-S6279.1)	Milyang23/SNUSG1 F_2:7_ RIL	Lim et al., 2014
5	*qFG3.1*	RM4996-RM7	1.3	*qFGP3a* (RM135-168)	Xieqingzao B/Zhonghui 9308 RIL	Feng et al., 2015
				*qFGN3* (RM85-RM227)	Ce258/IR75862 (IL)	Zhang et al., 2018
6	*qTN6.1*	RM276-RM204	3	*qTN6.1* (RM3)	Milyang23/SNUSG1 F_2:7_ RIL	Lim et al., 2014
7	*qPW3.1*	RM4996-RM7	1.3	*qpw3.2* (RM85)	Swarna/*O. nivara* BIL population	Haritha et al., 2018
8	*qCL6.1*	RM19291-RM217	3.01	*qCL-6* (RM4447)	Hinohikari/WSS2//Hinohikari BC_1_F_1_ population	Sato et al., 2004

The QTL, *qSPY4.1*, with PV% 8.5 and LOD of 2.6, which was detected in this study along with Xing et al. (2014), Ma et al. (2016), and Haritha et al. (2018), reported grain yield QTLs on chromosome 4. Fan et al. (2019) also reported *qGYPP4* in the BIL population derived from *Oryza longistaminata.* In our study, 8 QTLs for biomass were identified on chromosomes 2, 4, 5, 7, and 12 with LOD values ranging from 3.96 to 10.15 with a maximum PV of 4.29%. Zhang et al. (2017) mapped the BM QTL on chromosome 12 in F_2:3_ and RILs population. Six QTLs, *qHI2.1, qHI2.2*, *qHI5.1*, *qHI5.2*, *qHI7.1*, and *qHI12.1*, were mapped with a maximum LOD of 3.7 and PV of 4.12% for *qHI2.1* QTL. In a previous study of Sabouri et al. (2009), they identified *qHI2* with 21.35% of phenotypic variance. Grain number of panicle is one of the most important traits to contribute to yield, with *qGN4.1* QTL identified as the highest additive effect QTL and maximum LOD value of 3.5. This QTL was previously reported by Xing et al. (2014), Kim et al. (2017), and Deng et al. (2017). Singh et al. (2018) also reported the same *qGN4.1* QTL in bi-parental RIL population.

The QTL *qFG3.1* was found with 9.6% of phenotypic variance. Feng et al. (2015) and Zhang et al. (2018) reported QTLs for filled grain numbers as *qFGP3a* and *qFGN3*, respectively. QTLs for unfilled grains per panicle were identified on chromosomes 1, 3, and 5 with LOD value of 10.0. Five QTLs were observed for UFG on chromosomes 1, 3, and 5 with different flanking markers in both the populations except for *qUFG3.1* (Rabiei et al., 2015), with 8.2% of phenotypic variance in F_2_ population. Two QTLs, *qTN3.1* and *qTN6.1*, for tiller number and *qPTN4.1* for productive tiller number, were identified. Lim et al. (2014) also reported the same QTL on chromosome 6 with LOD of 3.79. In the present study, *qPL4.1* was identified with different flanking markers in F_2_ and F_2:3_ with 8.1% of the phenotypic variance on chromosome 4 with above 2.5 LOD value. It was mapped by Lim et al. (2014) and Kim et al. (2017) in *indica-japonica* recombinant inbred line population. Xing et al. (2014) and Zhang et al. (2018) reported QTL for productive tiller number on chromosome 4. Zhang et al. (2018) reported *qPL4* QTL in the background of IL population derived from cross between ZGX1 (high-quality *indica* elite variety) × IR75862 (high iron and zinc *japonica* variety).

Among the QTLs detected, the plant height QTL, *qPH1.1*, was identified in F_2:3_ with 13% of phenotypic variance value and with LOD value of 3.5. The same flanking marker RM246 for plant height QTL was previously reported by Zhang et al. (2010) in F_2_ population derived from cross between PA64s (*indica*) × Nipponbare (*japonica*) and Rabiei et al. (2015) in F_2:4_ population derived by crossing two *indica* rice varieties (Sepidrood × Gharib). Sabouri et al. (2009) also identified the same plant height QTL in F_2_ and F_2:3_ populations derived by crossing two genetically divergent *indica* type high-yielding rice varieties. Qiao et al. (2016) reported *qPH1.1* and *qPH1.2* with 7.01 and 9.05% PV, respectively, in the 198 CSSLs population derived from cross between *indica* var. 9311 and wild species *O. rufipogon* as donor parent. QTL *qPH1.1*, in BILs/ILs population previously derived from Swarna/*O. nivara* but with different flanking markers in chromosome 1, was reported by Swamy et al. (2014), Surapaneni et al. (2017), Haritha et al. (2018), and Balakrishnan et al. (2020). Another plant height QTL was mapped in F_2_ population with 3.7 LOD with a phenotypic variance of 12.5% on chromosome 6. *qPH6.1* QTL was previously reported in the F_2_ mapping population (Zhang et al., 2010) and in F_2:7_ recombinant inbred line population derived from a cross between Xiaobaijingzi (upland rice) and Kongyu 131 (Xing et al., 2014). Lim et al. (2014) also identified plant height QTL on chromosome 6 in F_2:7_ RIL population by crossing *indica* × *japonica* (Milyang23/SNUSG1) using the QGene 4.3.10 software.

*qFLL6.1* was identified with phenotypic variance of 7.4% and LOD value of 2.8 in the present study with the flanking region between RM217 and RM402. RM402 was detected in the same region in the study reported by Shen et al. (2011) who identified *qFLL6.1* and *qFLL6.2* in the 4.2 Mb region between flanking markers RM4923 and RM402 on short arm of chromosome 6 in NILs. Shao et al. (2009) reported flag leaf length QTL on chromosome 6. Matsubara et al. (2016) and Yang et al. (2018) reported *qFLL* QTL on chromosome 6 in RIL population and DH population, respectively. Leaf length QTL *qLL9.1* was identified with phenotypic variance of 6.9% with LOD of 2.6, while two QTLs for leaf width, *qLW9.1*, *qLW1.1*, were identified with 11 and 5.7% phenotypic variance on chromosome 9 and 1, respectively.

Seven QTLs for days to initial flowering were identified on chromosomes 1, 4, 7, 10, and 12. The QTL with the highest PV of 8.65% was observed with LOD value of 4.84 on chromosome 7. Mohammadi et al. (2013) reported QTLs for number of days to flowering on chromosomes 4 and 10 in the F_2_ population of Sadri/FL478 cross under saline field conditions. The major effect heading date QTLs was previously identified in chromosome 7 by Li et al. (2003) in IR64/Azucena doubled-haploid population. Yano et al. (2000) and Xue et al. (2008) identified *Hd1* on chromosome 6 and *Ghd7* on chromosome 7. Salgotra et al. (2015) reported *Ghd7* gene with associated traits of grains per panicle and plant height along with heading date. These previous reports also confirmed detection of the major QTLs in the same chromosomal regions as identified in our study.

### Consistent Quantitative Trait Loci Detected in This Study

*qTGW8.1* is a novel QTL consistently identified within the flanking marker region between RM3480 and RM3452 in three populations. *GW8.1* was previously mapped from another wild species *O. rufipogon* (Tian et al., 2006; Kang et al., 2018), and QTL for panicle weight was also reported from *O. nivara* on chromosome 3 but with different flanking markers. The number of traits studied was different across the generations and only 8 traits were phenotyped in all three generations. Therefore, we could check the consistency of QTLs of only these 8 traits. Further, we checked the consistency for the selected QTLs above threshold level after 1,000 permutations so QTLs below the threshold level, even in one generation, were not considered consistent even though the QTLs were mapped. As the QTL mapping was carried out in different generations, reduction in segregation, heterozygosity, change in context (background genome), and further stabilization of lines and environmental factors may also have contributed to identification of only few consistent QTLs. Stable QTLs which are consistently detected in different generations are useful in marker assisted selection (MAS) for crop improvement (Guo et al., 2005; Su et al., 2010).

### Quantitative Trait Loci for Grain Weight and Further Genetic Dissection

Of all the major QTLs detected, *qTGW8.1* was mapped in all three generations of F_2_, F_2:3_, and F_2:4_ at the same marker interval of RM3480-RM3452, covering a 2.6 Mb region with significant PVE ranging from 6.1 to 9.8%. Previous studies showed *qTGW* QTL in the background of *O. nivara* parent on other chromosomes 1, 2, 4, and 5 by Kaladhar et al. (2008), on chromosomes 1, 2, 3, and 4 by Swamy et al. (2014), and on chromosomes 1, 4, and 5 by Haritha et al. (2018). *qTGW8.1*, identified in the present study, is a novel QTL with *O. nivara* contributing to the additive effect. This region is already reported to harbor genes, *viz.*, Wealthy Farmer’s Panicle (WFP) encoding OsSPL14^WFP^ (Miura et al., 2010) and Ideal Plant Architecture (IPA1) encoding OsSPL14 (Jiao et al., 2010) and located 875 kbp upstream of already reported QTL *qGW8*, with gene Os08g0531600 (*GW8*/OsSPL16) (Wang et al., 2012). The identified QTL in this study could be a novel allele of these reported genes from *O. nivara* or a novel gene/QTL colocated in this region since there is an overlap with three known genes. However, this requires further genetic dissection and confirmation. The QTL trait enhancing allele is from the male parent 14s. Gel pictures of these markers showed a lower band size at 14s compared to Swarna which is indicative of the presence of a novel allele with deletion at the locus RM3480 triggered by recombination or any unknown factors contributed by *O. nivara* or Swarna genome.

Three meta-QTLs for yield on chromosome 8 (MQTL 8.1, 8.2, 8.3) were detected for various yield and grain-related traits. MQTL8.2 (326 kb) was identified as a recombination hot spot suitable for fine mapping yield-related traits including grain weight (Swamy and Sarla, 2011). It is interesting to note that the only meta-QTL for TGW on chromosome 8 (MQTL-GW4 S3680-RM3689 at 18.25–19.33 Mb), reported recently (Wang et al., 2021), is proximal to our *qTGW8.1*. The identified QTL in this study could be a novel allele of these reported genes from *O. nivara* or a novel gene colocated in this region. As the trait enhancing QTL allele is from male parent 14s and the gel pictures of these markers showed a lower band size at 14s compared to Swarna, it indicates the presence of a novel allele with possible deletions in 14s at loci RM3480, RM3452, and RM23447 compared to Swarna and can be confirmed with sequencing.

Thousand-grain weight QTL, detected in an advanced back cross between Nipponbare as recipient and Xihui 18 as the donor parent, was linked with RM6845. In addition, it is 169.5 kb away from RM3480, the flanking marker of *qTGW8.1* in the present study. *qGL-8*, a novel QTL for grain length, was identified at 22.70–22.05 Mb region at chromosome 8 using a set of recombinant inbred sister lines and high-yielding hybrid rice variety Nei2You No. 6 (Kang et al., 2021). Three QTLs for grain size-related traits, viz., *qGL8* (RM408–RM3702), *qLWR8*, and *qGT8* (RM3845–RM6948), were identified in chromosome 8 in F_2_ and F_2:3_ populations derived from an introgression line ‘IL188’ of Nipponbare/*Oryza minuta* cross (Feng et al., 2021). Thus, this consistent effect region on chromosome 8 seems important for several grain traits and is worthy of further fine mapping, cloning, and functional analysis, similar to that shown in a recent study using CSSLs (Wang et al., 2021). Most of the previous reports detected grain weight QTL in different genomic region compared to *qTGW8.1* of our study which is located between 24867542 to 27481361bp region [QTARO database (affrc.go.jp)] at marker interval of RM3480-RM3452, covering a 2.6 Mb region in the long arm of chromosome 8.

Another TGW QTL, *qTGW6.1*, was mapped in both F_2:3_ and F_2;4_ with LOD value of 2.51–3.52 (F_2:4_) and PV of 9.33–15.19% (F_2:4_) in the present study. Ishimaru (2003) reported that the *qTGW6.1* in backcross inbred lines was derived from cross between *japonica* (Nipponbare)/*indica* (Kasalath) after over 3 years of testing. *qTGW6.1*, at marker interval of RM402- RM5963, was detected in the IL population of ZGX1 × IR75862 (Zhang et al., 2018). One of the flanking markers of this QTL RM3452 was reported to be linked with grain width in the F_2_ population of Kasalath × BG23 (Segami et al., 2016) and was also associated with leaf traits (Yang et al., 2018). Zhu et al. (2019) also identified thousand-grain weight QTLs on chromosomes 6 and 8 in three recombinant inbred line populations of Teqing/IRBB lines (TI), Zhenshan97/Milyang46 (ZM), and Xieqingzao/Milyang46 (XM).

### Quantitative Trait Loci Clusters Detected

In this study, several QTL clusters were detected, and some of the clusters contained more than four QTL, signifying a heavily populated region of QTL on the chromosome. Xie et al. (2008) detected a cluster of yield-related QTLs on chromosome 9, in BC_3_F_4_ population from an interspecific cross of Hwaseong/*O. rufipogon* (IRGC 105491). Jeon et al. (2021) employed map-based cloning, gene sequencing, expression analysis, and transgenic approaches to detect putative genes and the genetic architecture of the cluster. The study using BC_4_F_4_ NILs of the same population demonstrated that the cluster is regulated by a single pleiotropic gene (*ascorbate peroxidase* gene, APX9). Multiple traits mapping in the same region of a chromosome are beneficial to breeders, as QTL clusters allow breeders to focus their efforts on regions of the genome containing the most QTLs of interest. The identification of gene clusters is useful in marker-assisted selection since the markers delineating these regions can be chosen for selecting the traits of interest. Our study demonstrated that favorable alleles from stable wild introgression lines with minimum linkage drag can be introduced to develop high-yielding lines.

## Conclusion

Inter-specific crosses are bridging tools to introgress beneficial alleles from wild donor into cultivar background to get improved lines for breeding and cultivation. Alien introgression lines/BILs were used as a crossing material in this study, which is a novel approach to detect QTLs and is further useful for QTL/gene pyramiding. In this study, 21, 30, and 17 QTLs were mapped in F_2_, F_2:3_, and F_2:4_, derived from a two BILs for a total of 20, 18, and 8 yield-related traits, respectively. This breeding strategy helped to converge beneficial alleles from both the parents to produce superior lines. Five lines, C_2_12, C_2_124, C_2_128, C_2_143, and C_2_162 were significantly higher yielding than Swarna and 14s and carried alleles for *qTGW8.1* from *O. nivara* in either a homozygous or heterozygous condition. The F_2_, F_2:3_, and F_2:4_ QTL mapping helped to identify 8 major QTLs, namely, *qPH1.1*, *qPH6.1*, *qTGW6.1*, *qTGW8.1, qTN6.1, qPW3.1, qCL6.1*, and *qFG3.1* for yield-related traits. *qTGW8.1* is found as a potential candidate region of 602.4 kb for genetic dissection. The significantly different lines with major yield QTLs will be utilized in further fine mapping and identification of genes for yield improvement. Populations generated from these BIL/CSSL are useful to detect the candidate gene location for important agronomical traits by further fine mapping. This strategy of intercrossing advanced back cross introgression lines can be employed in other backgrounds with advanced genomics tools for crop improvement programs. The statistically significant lines identified by pair-wise mean comparison with both the parents and their common cultivar parent Swarna is useful in gene discovery for target traits and to mine possibly better alleles from the wild progenitors for further crop improvement.

## Data Availability Statement

The original contributions presented in the study are included in the article/Supplementary Material, further inquiries can be directed to the corresponding author/s.

## Author Contributions

SN and DB conceptualized, designed, and supervised the experiments, and contributed to the final revision of the manuscript. KB performed the experiments. KA, MS, and VR supported conducting the field experiments. KB and DB analyzed the data and wrote the manuscript with contributions from SN. All authors contributed to the article and approved the submitted version.

## Conflict of Interest

The authors declare that the research was conducted in the absence of any commercial or financial relationships that could be construed as a potential conflict of interest.

## Publisher’s Note

All claims expressed in this article are solely those of the authors and do not necessarily represent those of their affiliated organizations, or those of the publisher, the editors and the reviewers. Any product that may be evaluated in this article, or claim that may be made by its manufacturer, is not guaranteed or endorsed by the publisher.

## Footnotes

1
http://bbi.irri.org/products

2
http://www.isbreeding.net
