# Supplementary material for: Mapping of QTLs for Yield Traits Using F2:3:4 Populations Derived From Two Alien Introgression Lines Reveals qTGW8.1 as a Consistent QTL for Grain Weight From Oryza nivara
Source: Front Plant Sci. 2022 Mar 9;13:790221. doi: 10.3389/fpls.2022.790221 (PMC8959756; doi:10.3389/fpls.2022.790221)
Supplement: Supplementary file 1 [file Data_Sheet_1.doc]

**Mapping of QTLs for yield related traits using F_2:3:4_ populations derived from two alien introgression lines reveals *qTGW8.1* as a consistent QTL for grain weight from *Oryza nivara***

**Kavitha Beerelli**^1,2^**, Divya Balakrishnan**^1*^, **Krishnam Raju Addanki**^1,2^, **Malathi Surapaneni**^1^, **Venkateswara Rao Yadavalli**^1^, **Sarla Neelamraju**^1*^

^1^ICAR-Indian Institute of Rice Research, Hyderabad-500 030, Telangana, India

^2^Acharya Nagarjuna University, Guntur-522 510, Andhra Pradesh, India

[******sarla_neelamraju@yahoo.com*](mailto:*sarla_neelamraju@yahoo.com)*,* [**dbiirr23@gmail.com*](mailto:*dbiirr23@gmail.com)

| **Supplementary Fig 1. Frequency distribution of 19 yield related traits in 166S x 14S F_2_ population** | | |
| --- | --- | --- |
| 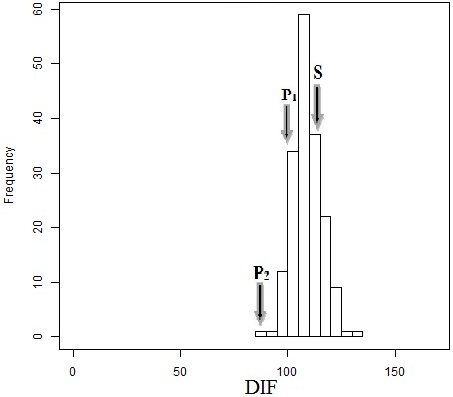 | 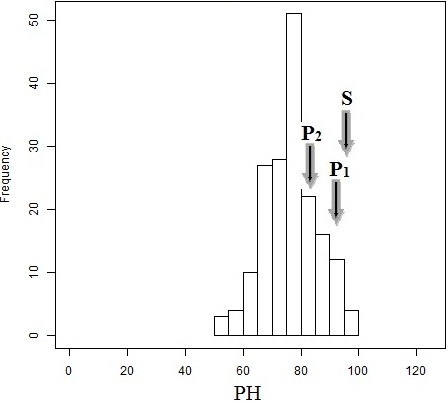 | 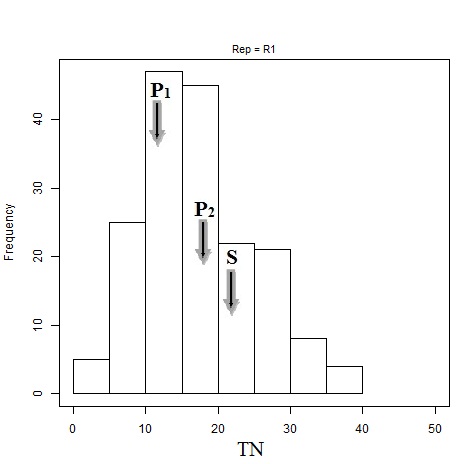 |
| 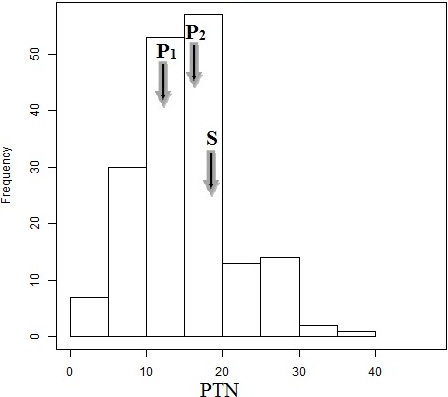 | 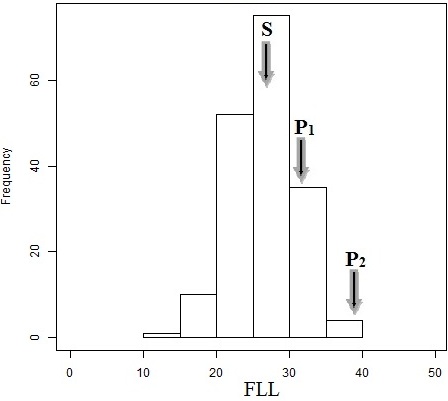 | 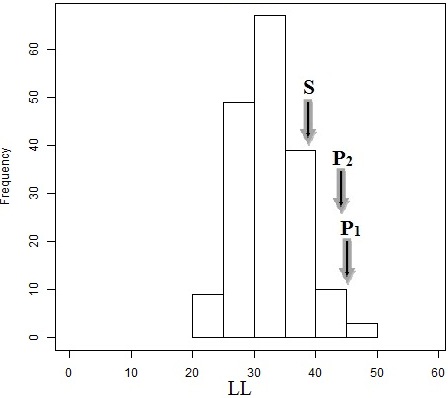 |
| 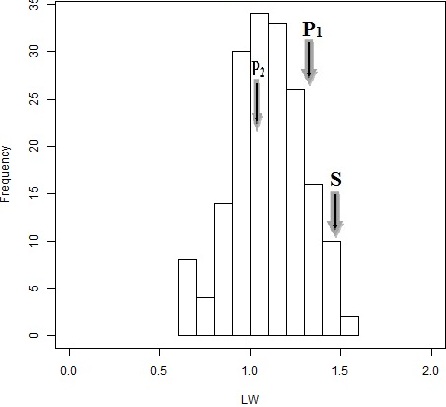 | 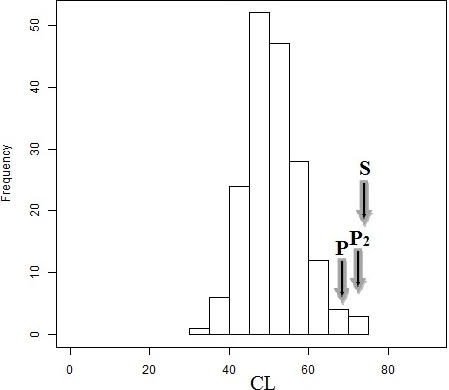 | 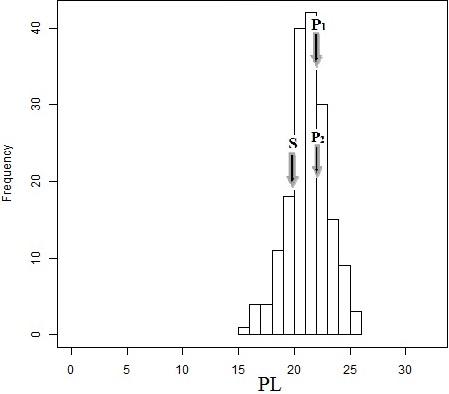 |
| 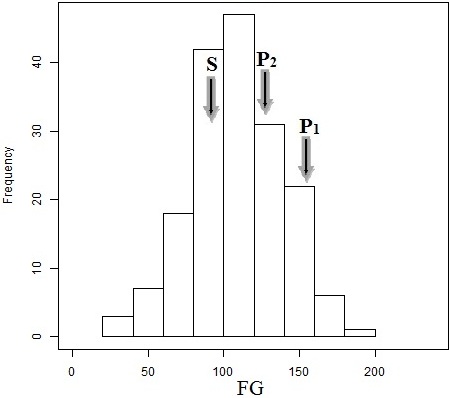 | 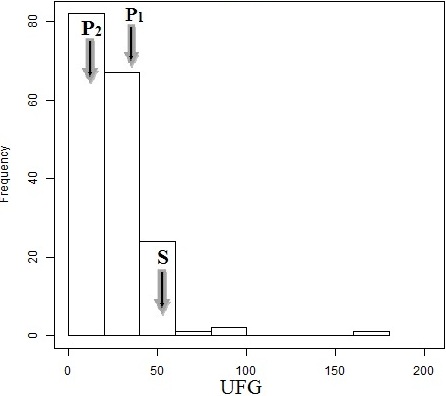 | 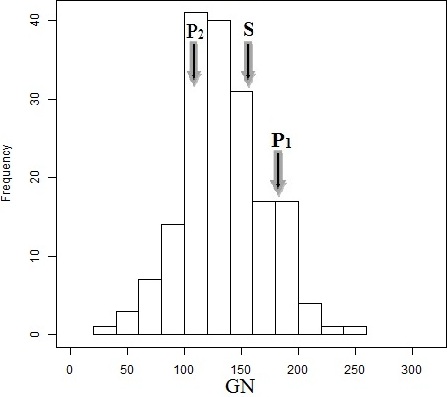 |
| 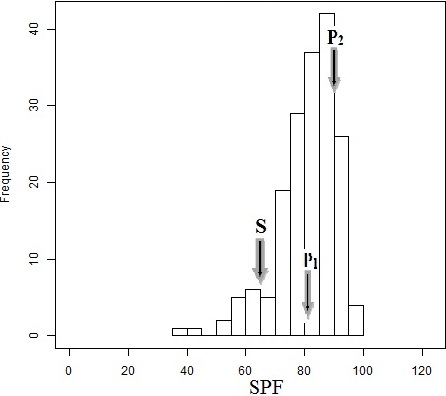 | 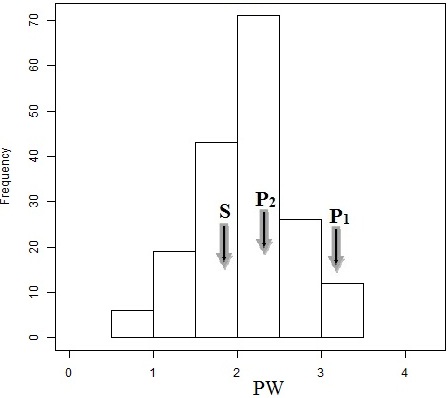 | 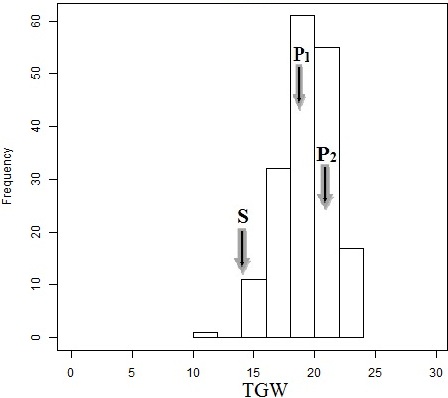 |
| 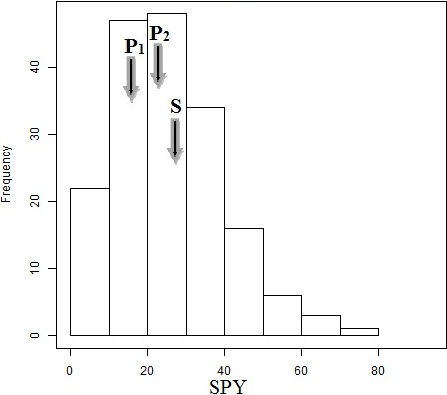 | 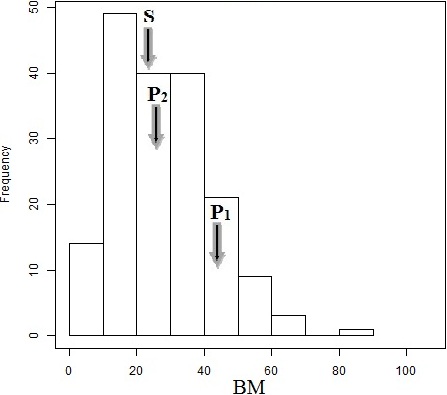 | 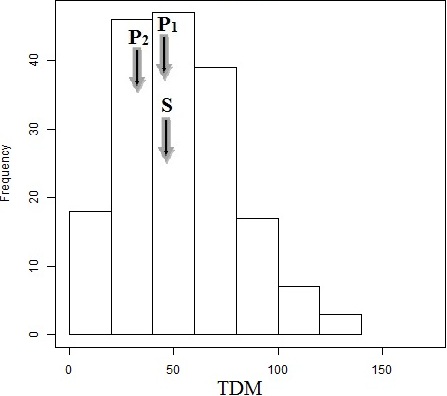 |
| 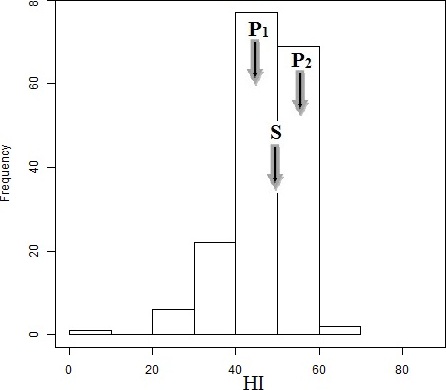 | | |

P_1_-166S, P_2_-14S, S- Swarna, DIF- days to initial flowering, PH- plant height, TN- Tiller number, PTN- Productive tiller number, FLL- Flag leaf length, LL- Leaf length, LW- Leaf width, CL-Culm length, PL- Panicle length, FG- Filled grains, UFG- Unfilled grains, GN- Grain number, SPF- Spikelet fertility, PW-Panicle weight, TGW-Thousand grain weight, SPY- Single plant yield, BM- Biomass, TDM- Total dry matter and HI- Harvest index.

| **Supplementary Fig 2: Frequency distribution of 18 yield related traits in F_3_ population of 166S x 14S** | | |
| --- | --- | --- |
| 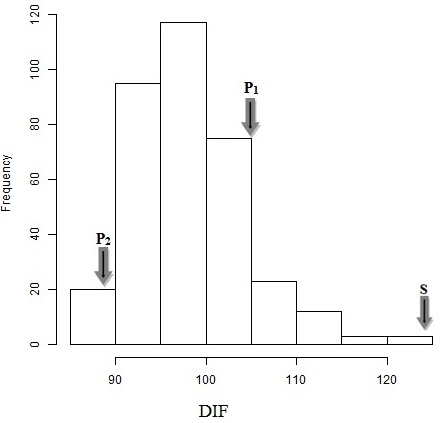 | 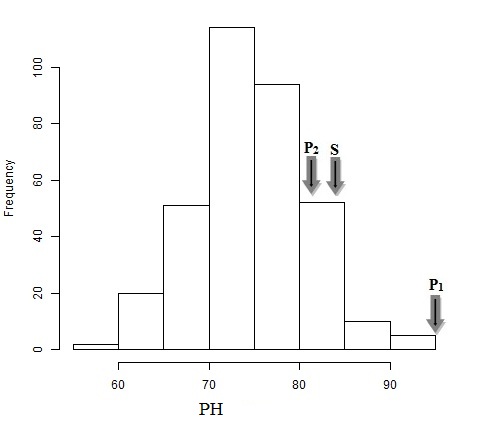 | 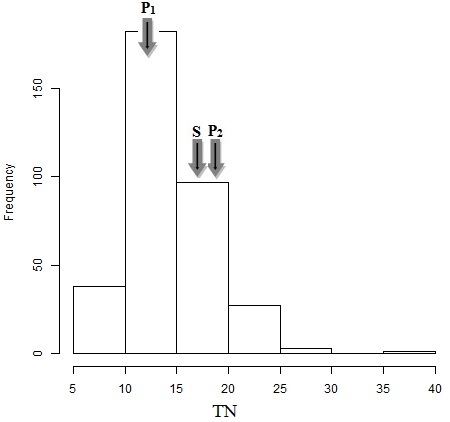 |
| 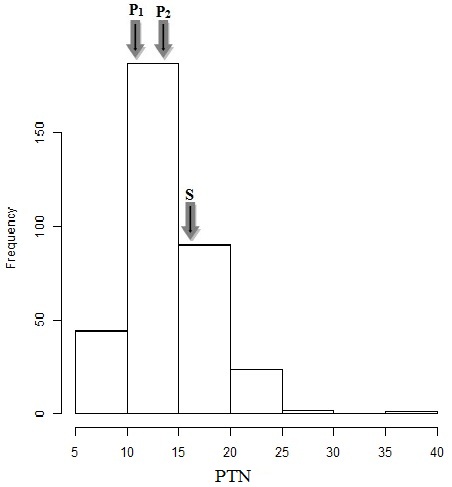 | 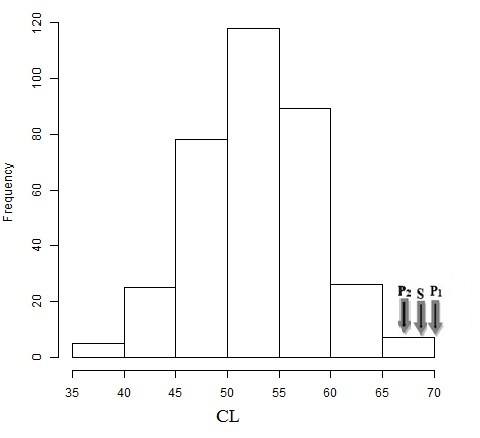 | 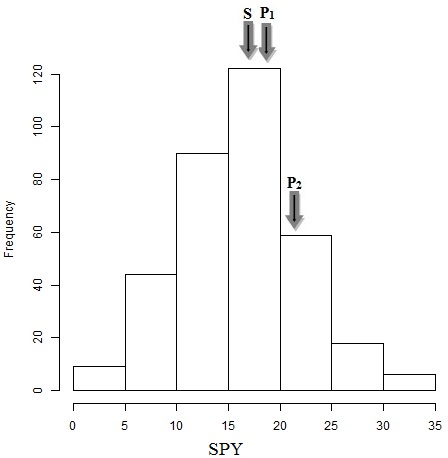 |
| 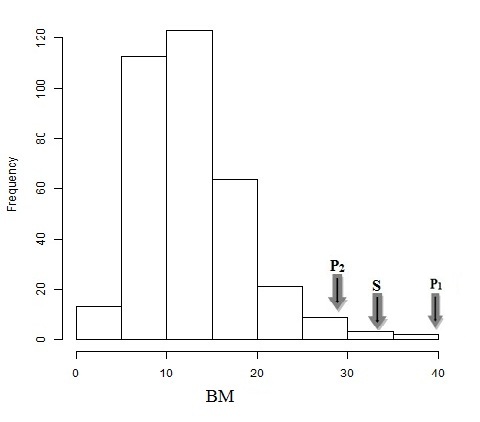 | 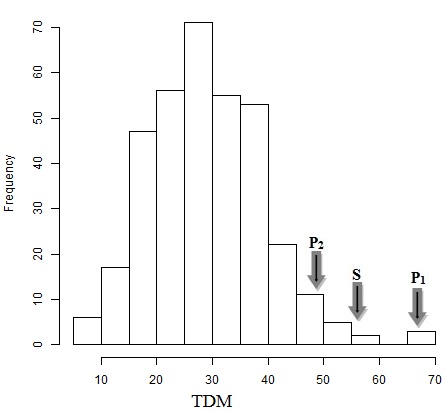 | 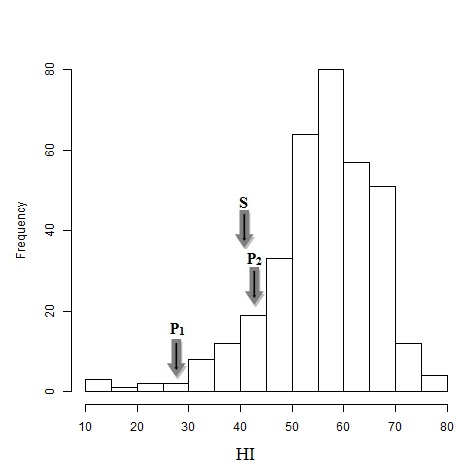 |
| 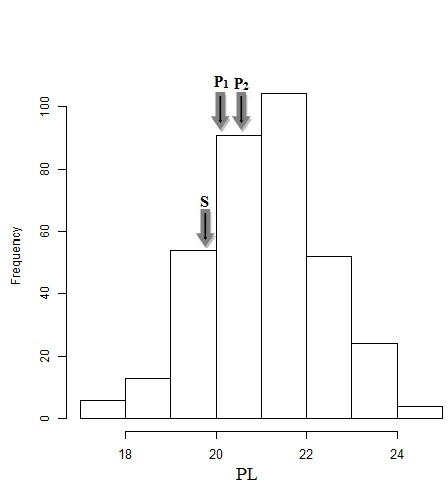 | 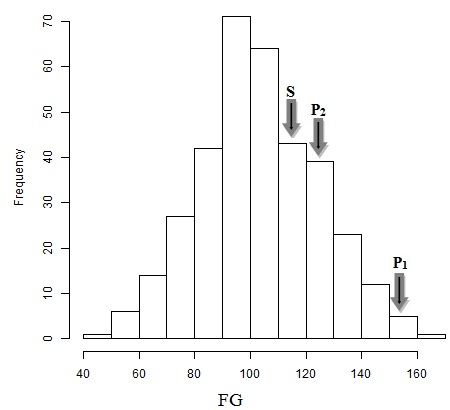 | 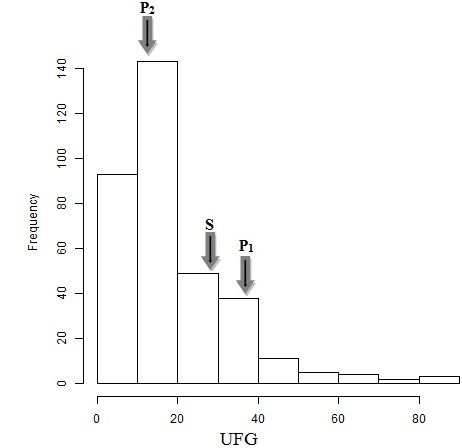 |
| 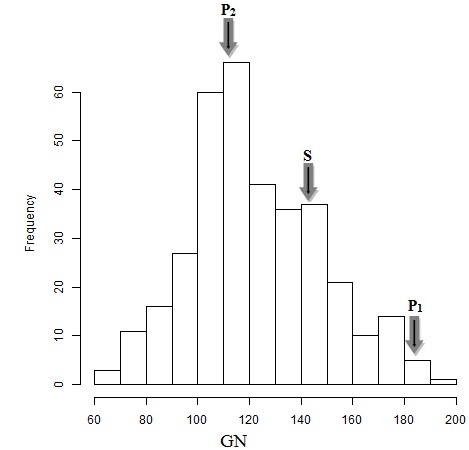 | 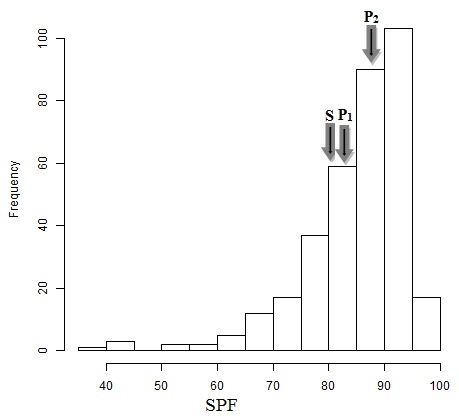 | 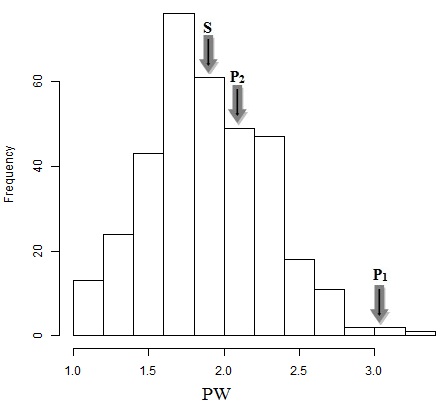 |
| 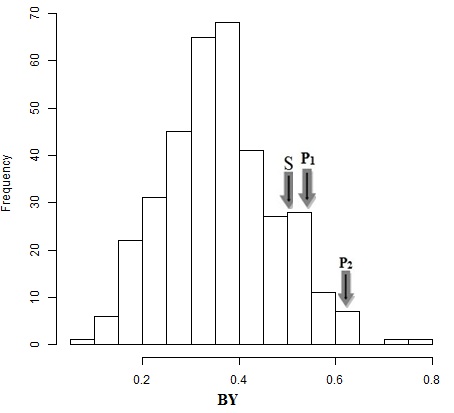 | 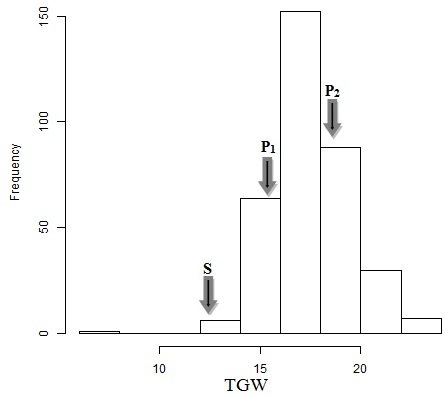 | 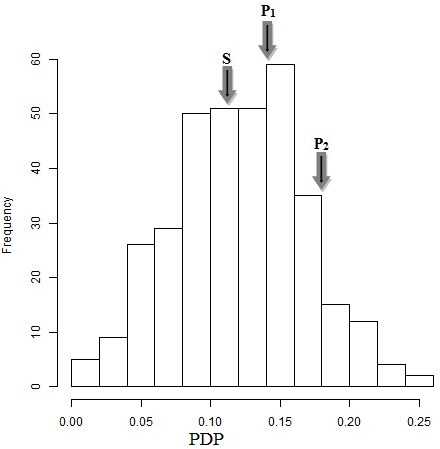 |

P_1_-166S, P_2_-14S, S- Swarna, DIF- days to initial flowering, PH- plant height, TN- Tiller number, PTN- Productive tiller number, CL-Culm length, SPY- Single plant yield, BM- Biomass, TDM- Total dry matter, HI- Harvest index, PL- Panicle length, FG- Filled grains, UFG- Unfilled grains, GN- Grain number, SPF- Spikelet fertility, PW-Panicle weight, BY- Bulk yield, TGW-Thousand grain weight and PDP- Per day productivity.

| **Supplementary Fig 3. Frequency distribution of 8 yield related traits in F_4_ population of 166S x 14S** | | |
| --- | --- | --- |
| 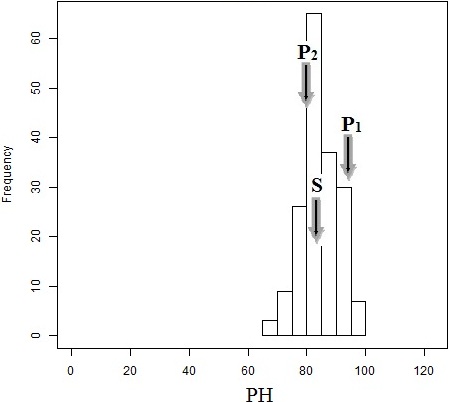 | 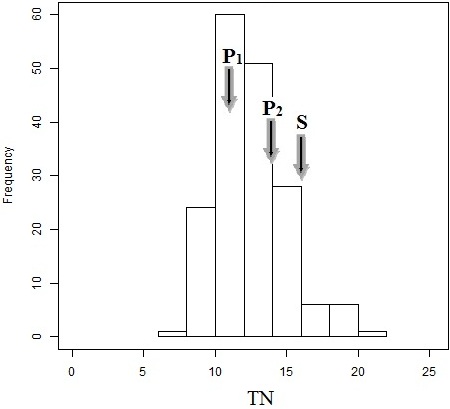 | 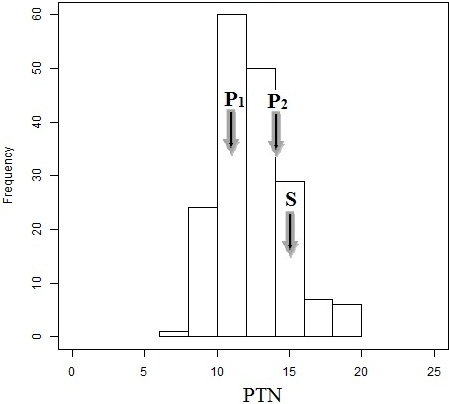 |
| 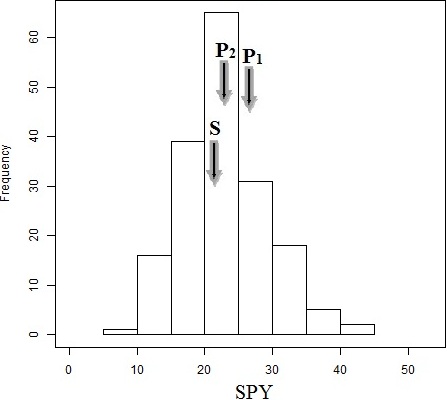 | 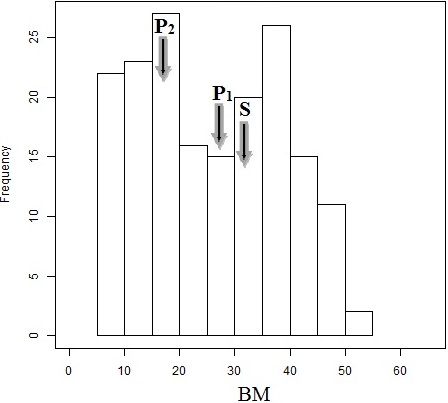 | 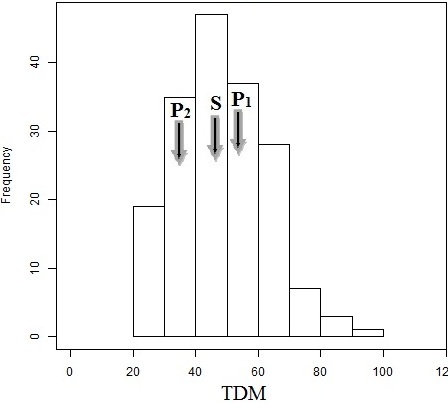 |
| 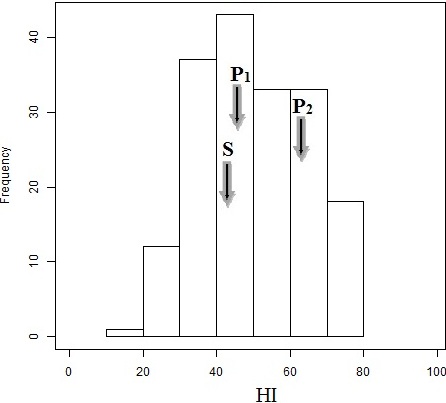 |  | 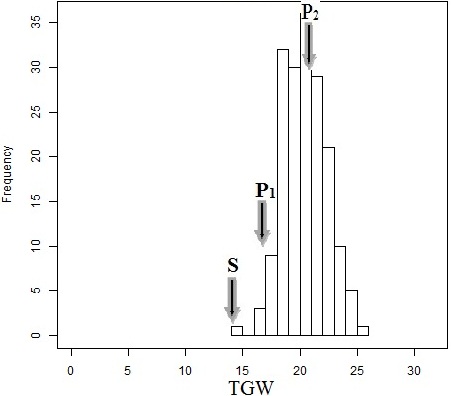 |
| P_1_-166S, P_2_-14S, S- Swarna, PH- plant height, TN- Tiller number, PTN- Productive tiller number, SPY- Single plant yield, BM- Biomass, TDM- Total dry matter, HI- Harvest index and TGW-Thousand grain weight. | | |

| **Supplementary Fig 4. Box plots of 8 common yield related traits in F_2_, F_3_ and F_4_ populations of 166S x 14S** | | |
| --- | --- | --- |
| 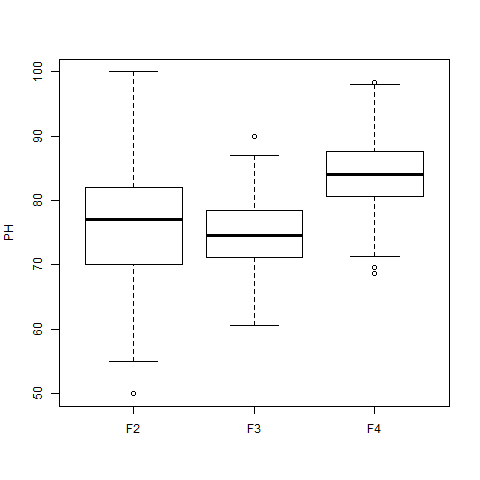 | 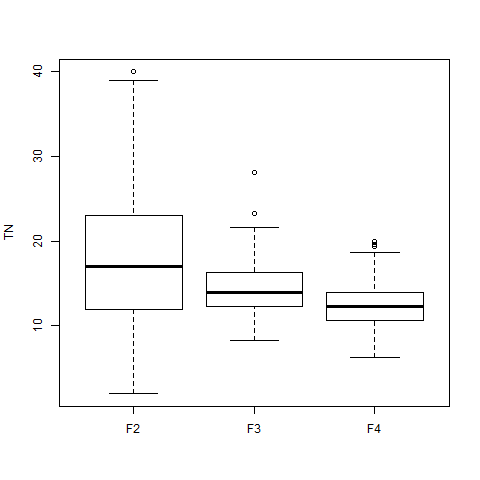 | 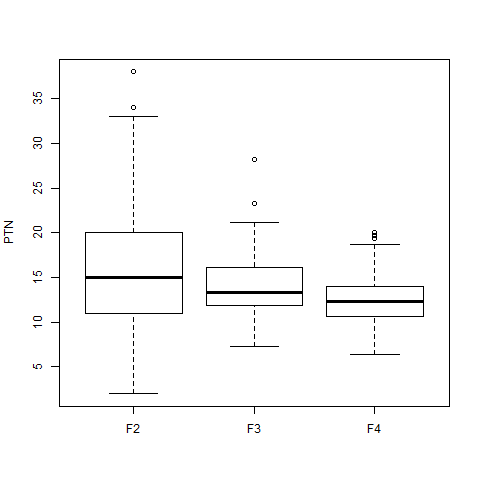 |
| 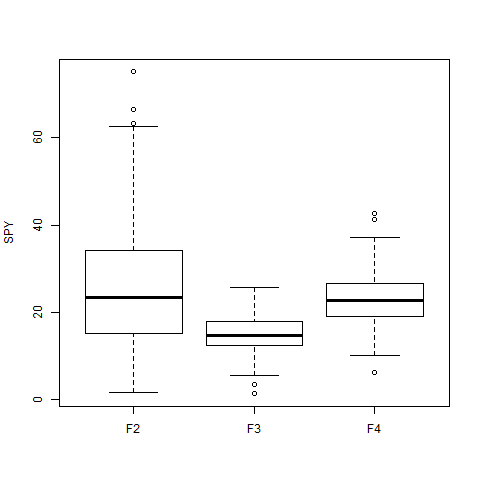 | 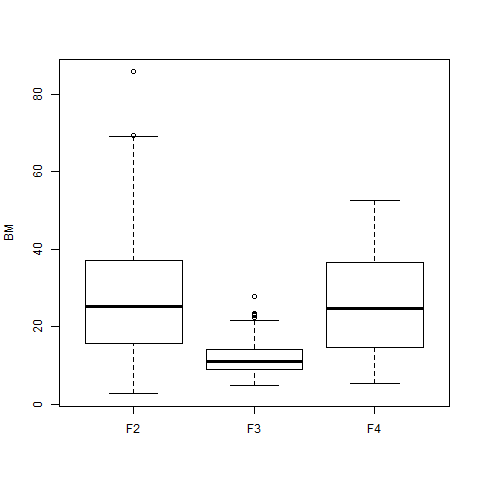 | 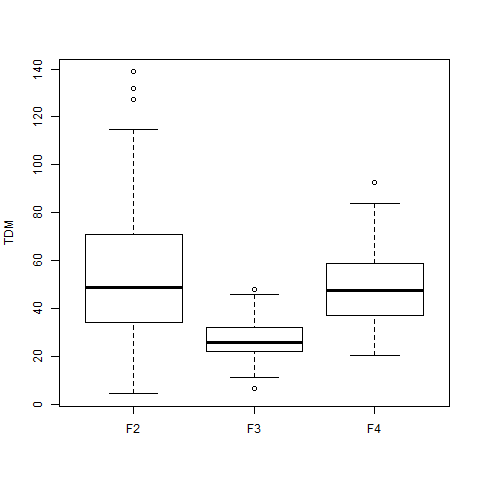 |
| 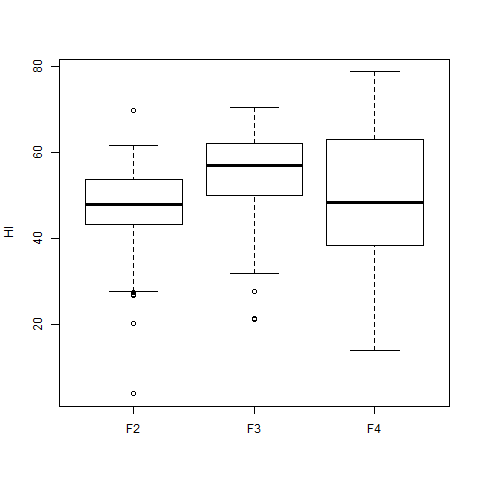 |  | 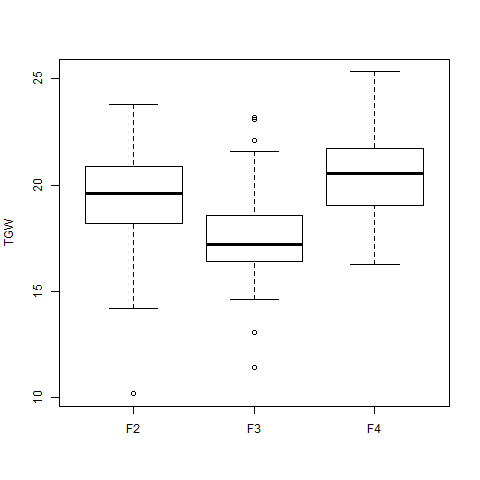 |

| F_2_, F_3_ and F_4_-Generations, PH- plant height, TN- Tiller number, PTN- Productive tiller number, SPY- Single plant yield, BM- Biomass, TDM- Total dry matter, HI- Harvest index and TGW-Thousand grain weight. |
| --- |

| **Supplementary Fig 5. Box plots of 16 yield related traits in F_2_ and F_3_ populations of 166S x 14S** | | | |
| --- | --- | --- | --- |
| 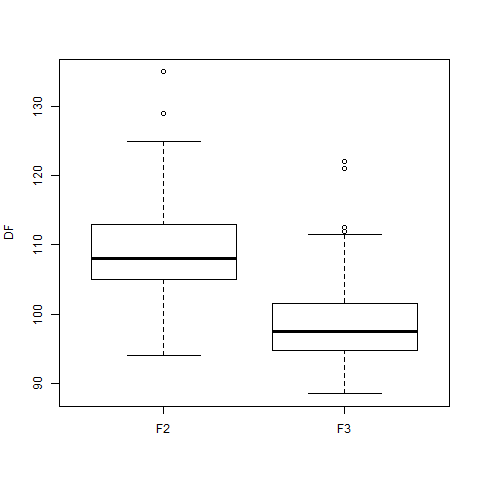 | 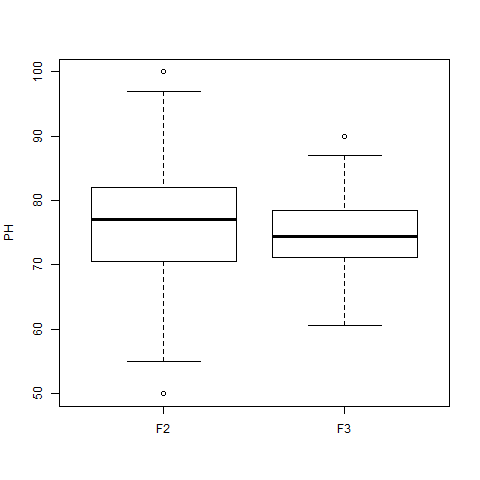 | | 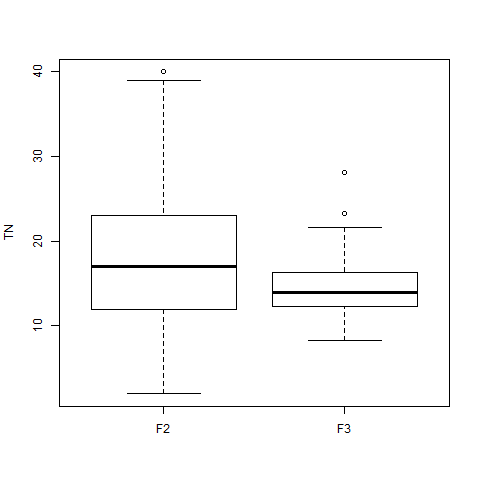 |
| 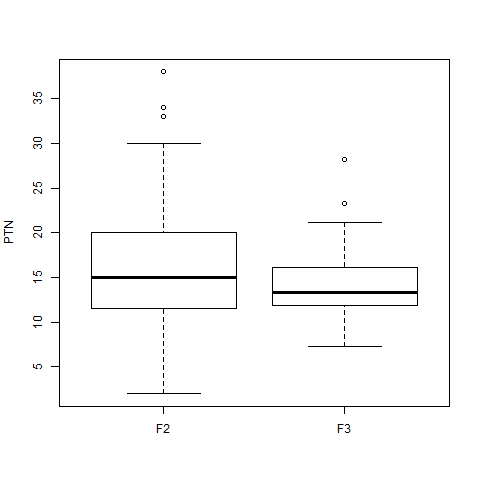 | 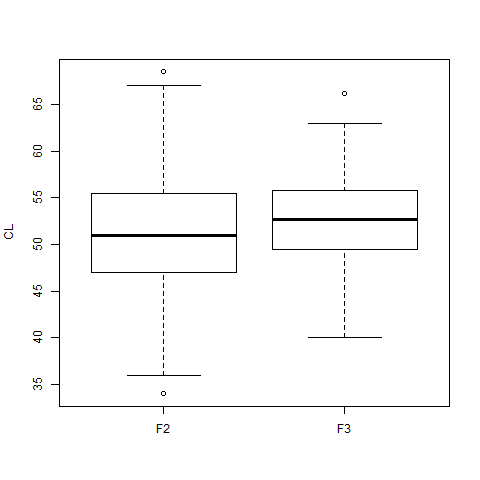 | | 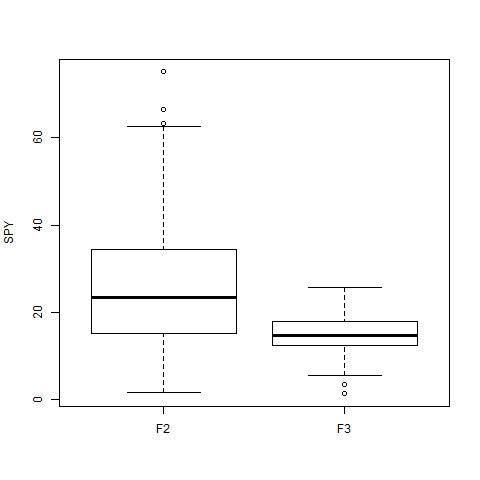 |
| 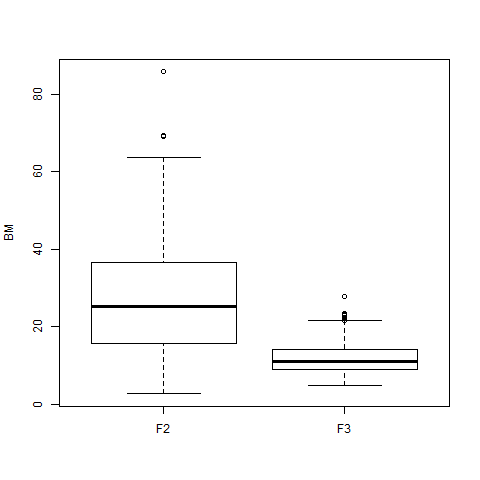 | 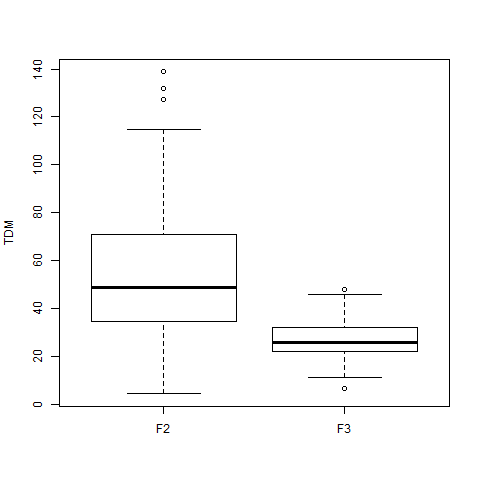 | | 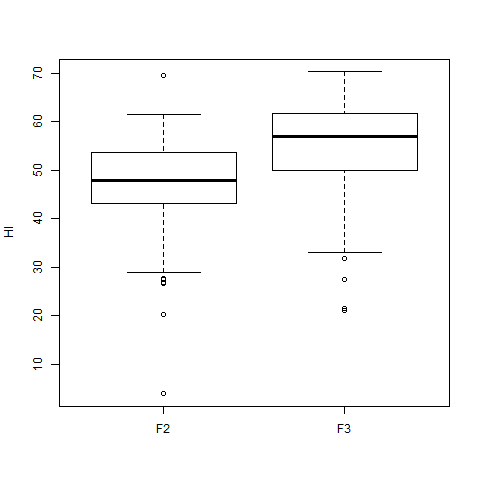 |
| 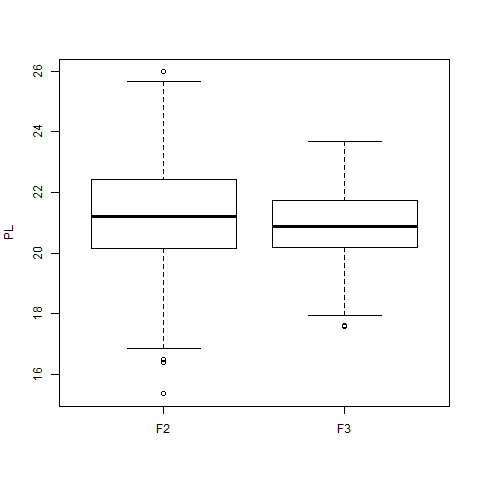 | 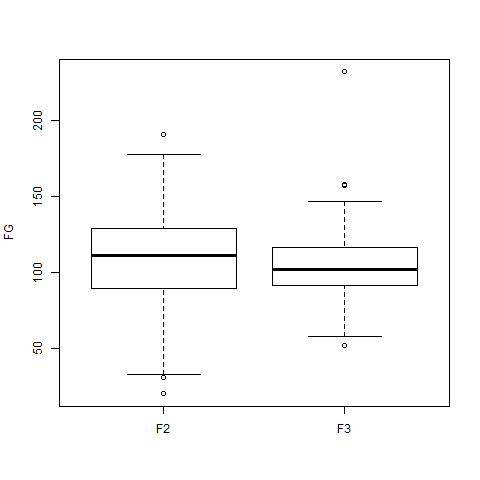 | 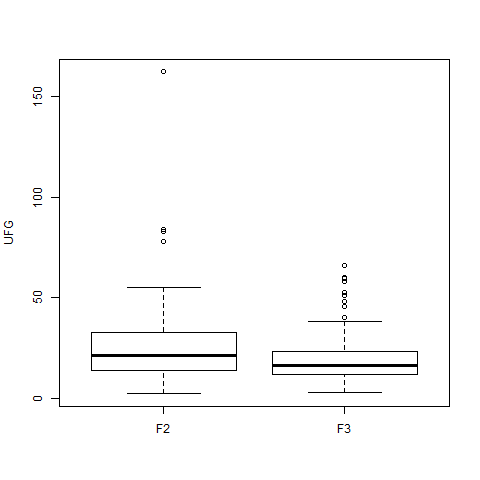 | |
| 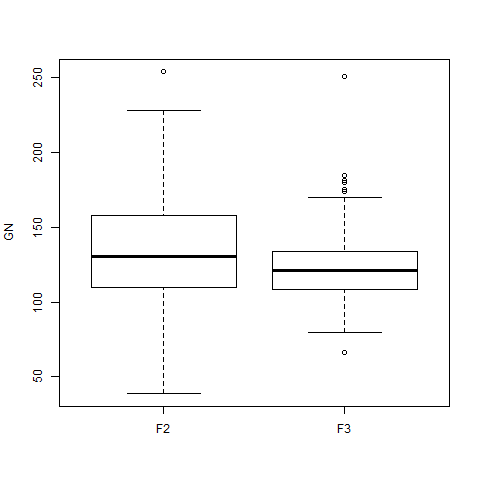 | 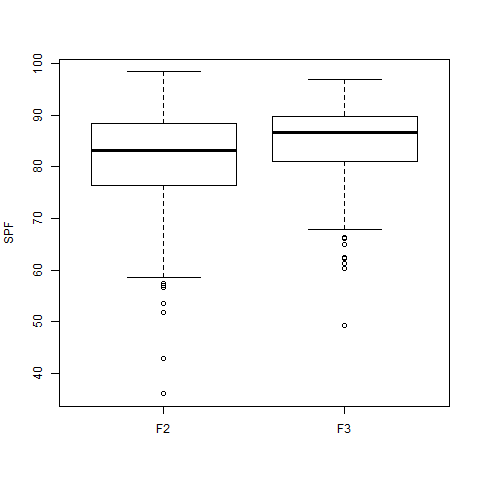 | 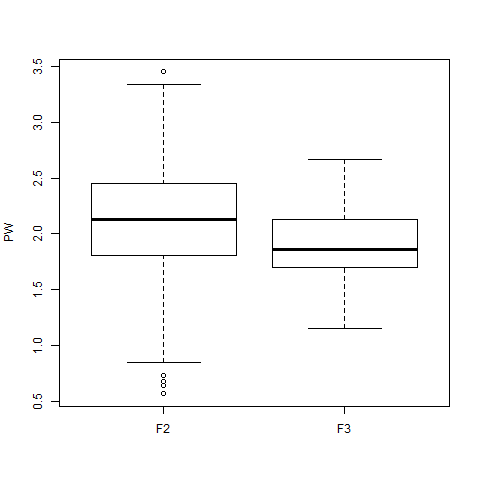 | |
|  | 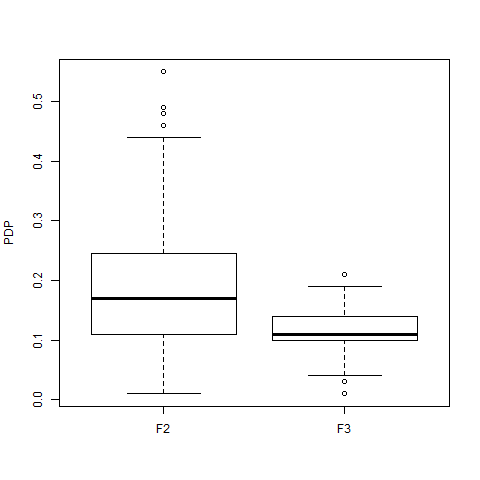 |  | |

F_2_, F_3_-Generations, DIF- days to initial flowering, PH- plant height, TN- Tiller number, PTN- Productive tiller number, CL-Culm length, SPY- Single plant yield, BM- Biomass, TDM- Total dry matter, HI- Harvest index, PL- Panicle length, FG- Filled grains, UFG- Unfilled grains, GN- Grain number, SPF- Spikelet fertility, PW-Panicle weight and PDP- Per day productivity.
